# New nicotine and tobacco products and risk of major diseases: A review of the scientific evidence

**DOI:** 10.1177/14034948251391690

**Published:** 2025-11-12

**Authors:** Sofia Carlsson, Miranda Beck, Anna Bergström, Kristian Dreij, Koustav Ganguly, Maria Kippler, Federica Laguzzi, Karin Leander, Donghau Lu, Lena Palmberg, Swapna Upadhyay, Anna Zettergren, Göran Pershagen

**Affiliations:** Institute of Environmental Medicine, Karolinska Institutet, Stockholm, Sweden

**Keywords:** E-cigarette, nicotine pouches, heated tobacco products, health, diabetes, lung, cardiovascular, cancer, asthma, pregnancy

## Abstract

The use of new tobacco and nicotine products, including e-cigarettes, heated tobacco products (HTPs) and tobacco-free snus (white snus), is rising, especially among adolescents and young adults. However, evidence on their long-term health effects is limited. This review assesses current knowledge on the health risks of these products, with a focus on asthma and allergic diseases, cancer, cardiovascular disease, diabetes, lung disease and pregnancy outcomes.

E-cigarettes are associated with increased risks of asthma, respiratory symptoms, chronic bronchitis and chronic obstructive pulmonary disease (COPD). Associations with cardiovascular disease, type 2 diabetes, cancer and adverse pregnancy outcomes are supported by limited epidemiological and experimental data. Less evidence is available on health risks associated with HTPs, but a few epidemiological studies indicate increased risks of asthma and COPD, with supporting data from experimental studies. Evidence on white snus is notably lacking across the different health outcomes. Given its high nicotine content – similar to or higher than in brown snus – it is plausible that white snus increases risks of diabetes, pregnancy complications and other nicotine-related health effects. Methodological limitations of existing epidemiological studies include reliance on self-reported data, inadequate adjustment for cigarette smoking, cross-sectional design and/or short follow up. Future research should prioritize large-scale, longitudinal studies assessing dose, duration and product formulation, including second-hand and prenatal exposure, as well as mechanistic studies. There is an urgent need to elucidate the health impacts of the new nicotine and tobacco products, which are growing in popularity, to inform policy, regulation and public health strategies.

## Introduction

The global burden of tobacco smoking has resulted in over 200 million premature deaths in the past 30 years, as well as many times more cases of acute and chronic disease [1]. In parallel, public health interventions and increased awareness of smoking hazards have led to declining smoking rates in many countries. However, the use of other tobacco products has remained prevalent in some countries, such as in Sweden, where snus continues to be widely used. Snus has also become increasingly popular in Norway in recent years.

Over the last decade, novel nicotine products such as electronic cigarettes (e-cigarettes), heated tobacco products (HTPs) and tobacco-free nicotine pouches (white snus) have gained popularity [2]. Particularly concerning is the rapid increase in use among adolescents, with flavours and marketing strategies targeted at younger individuals. In Sweden in 2024, 15.2% of women and 10.6% of men aged 16–29 reported daily use of white snus, and 13.0% and 7.4%, respectively, reported using e-cigarettes daily or occasionally [2]. E-cigarettes vapourize a liquid containing nicotine, propylene glycol/glycerine and flavourings. HTPs heat tobacco to release an aerosol, while white snus consists of flavoured nicotine pouches for oral use. All three product types deliver nicotine, a substance with high addictive potential. They are frequently promoted as being ‘tobacco free’, even though the nicotine found in e-cigarettes and white snus is typically derived from tobacco plants. Nicotine has been implicated in a range of adverse health effects, including cardiovascular, respiratory and reproductive disorders and may contribute to the development of type 2 diabetes [3]. Additionally, other constituents of these products, such as flavourings and solvents, may have toxic properties.

The emergence of new nicotine products presents a major challenge for public health, and the long-term health effects are poorly understood, as their widespread use is fairly recent. These products are often promoted as ‘harm reduction’ but are widely marketed to adolescents, many of whom have never smoked, creating a new group of nicotine users. Their efficacy in smoking cessation is poorly documented, and use may instead increase the risk of later cigarette smoking initiation. This review examines the current evidence on health risks associated with e-cigarettes, HTPs and white snus, focusing on outcomes of public health significance including asthma, cardiovascular disease (CVD), cancer, diabetes, lung disease and pregnancy outcomes. The aim is to update the understanding of their potential long-term effects on public health. This review is based on a recent comprehensive evaluation of the evidence by the Institute of Environmental Medicine at Karolinska Institutet [4].

## Methodology

### Literature review

We aimed to identify all relevant studies examining the health effects of new tobacco and nicotine products in relation to asthma and allergic diseases, cancer, CVDs, diabetes, lung conditions and pregnancy outcomes. Separate literature searches were conducted for each outcome, covering both epidemiological and experimental studies, including mechanistic studies in humans, animals or cell models. In July 2024, an information specialist at the Karolinska Institutet Library performed the searches using predefined criteria across four databases: Medline, Embase, Cochrane Library and Web of Science. No restrictions were applied regarding publication date or language. Full documentation of the search terms and strategies for each exposure and outcome is available [4]. This review does not address the concept of harm reduction, such as comparing the risks of new nicotine products with conventional smoking. Instead, the focus was on evaluating the health risks associated with the use of these products compared with no use of tobacco or nicotine products. Efforts were also made to exclude studies with financial ties to the tobacco industry or companies associated with production and promotion of the new nicotine products. To meet the length requirements, the review does not include the evidence on women’s health, which is covered in the full report [4]. Furthermore, a more extensive reference list is found in this report as well as in the Supplemental material.

## Asthma and allergic diseases

We identified 6 systematic reviews/meta-analyses on e-cigarette exposure, 77 epidemiological studies (70 on e-cigarettes, 5 on HTPs, 2 on both) and 14 mechanistic studies (Supplemental Tables 1–3).

### E-cigarettes

Several systematic reviews and meta-analyses have summarized epidemiological studies on e-cigarette use and allergic airway disease (Supplemental Table 1). In 2021, multiple reviews of primarily cross-sectional studies concluded that e-cigarette use was associated with asthma for both current and ever use. Pooled odds ratios (pORs) for ever use ranged from 1.24 to 1.39 [5-7]. Among adolescents, similar associations were found, with pORs of 1.36 (95% confidence interval (CI) 1.26–1.48) for current and 1.31 (95% CI 1.22–1.42) for ever use [8]. Another meta-analysis reported a pOR of 1.24 (95% CI 1.19–1.30) for current use and asthma or wheeze, with consistent results even among never-smokers [9]. A separate review found that the prevalence of wheeze among exclusive e-cigarette users was 19%, similar to dual users (21%) and those transitioning from smoking (17%) [10]. All reviews relied on self-reported exposure and outcome data from mostly cross-sectional designs, limiting causal interpretation. Further, it is unclear whether e-cigarette use leads to asthma onset or exacerbates existing disease.

Recent cross-sectional studies have largely supported earlier findings of a link between e-cigarette use and asthma symptoms, including among never-smokers (Supplemental Table 1). While several large studies from France and the US report increased risk, one Swedish study found no association, though it did not analyze wheeze separately. Longitudinal data are limited; however, in the Population Assessment of Tobacco and Health (PATH) cohort, current exclusive use was marginally associated with incident asthma (odds ratio (OR) 1.12, 95% CI 0.99–1.27) [11]. Further PATH studies found increased risk of asthma onset among young adults (hazard ratio (HR) 3.52, 95% CI 1.24–10.02) but not in never-smoking adolescents (Supplemental Table 1).

Evidence for other allergic outcomes is scarce (Supplemental Table 1). In Japan, e-cigarette use was more prevalent among men with atopic dermatitis, though not statistically significant [12]. In Korean adolescents, ever use was linked to asthma, atopic dermatitis and rhinitis; but only atopic dermatitis remained borderline significant in exclusive users [13]. In Korean adults, current use was associated with allergic rhinitis (OR 1.38, 95% CI 1.15–1.66), although only 1.5% of e-cigarette users were ever smokers [14]. In US adults, e-cigarette use was linked to atopic dermatitis overall (OR 1.35, 95% CI 1.16–1.58) and among never-smokers (OR 1.61, 95% CI 1.28–2.02), especially in women [15]. Parental e-cigarette use was associated with skin allergies in children (OR 1.24, 95% CI 1.08–1.42), including among non-smokers [16].

Mechanistic studies have focused on allergic airway responses (Supplemental Table 2). In humans, e-cigarette use increased airway matrix metalloproteinase-9, neutrophils and mucus proteins. In vitro, nicotine-free vapour increased inflammatory markers (interleukin-6, MUC5AC), suggesting that constituents beyond nicotine may contribute to airway disease mechanisms. Studies in mice have consistently shown that e-cigarette exposure contributes to asthma-like outcomes, including airway hyperresponsiveness, immune-cell infiltration, T-cell helper-2 cytokine production and mucus overproduction (Supplemental Table 2). The effects varied by flavour, nicotine content and base liquid (propylene glycol versus glycerine). Long-term exposure caused airway remodelling and sustained inflammation. Prenatal exposure in mice impaired lung development and increased allergic airway reactivity in offspring. One in-silico study identified flavouring agents with potential for skin sensitization.

### Heated tobacco products

Fewer studies have examined HTPs, but existing research suggests links to asthma and allergic diseases (Supplemental Table 3). HTP use has been associated with asthma in adolescents and adults, allergic rhinitis among adults and maternal use during pregnancy with allergic outcomes in children. Second-hand exposure has also been linked to asthma symptoms.

### Conclusions

The current literature indicates a consistent association between e-cigarette use and asthma and wheeze, supported by mechanistic data showing immune and airway changes. Evidence for other allergic diseases like dermatitis and rhinitis is sparse. Limited data suggest that prenatal and second-hand exposure to e-cigarettes and HTPs may also increase allergic disease risk in children.

Data on HTPs are emerging, with early studies indicating similar risks as for e-cigarettes, though lacking mechanistic support. Evidence on white snus is essentially non-existent.

Most studies rely on self-reported exposures and outcomes, with few prospective designs or clinical validations. Future research should prioritize longitudinal studies with objective measures and consider the influence of nicotine, flavours and dual use, that is, interactions with smoking. There is a need to study newer nicotine products besides e-cigarettes, especially in young and vulnerable populations.

## Cancer

We identified 15 epidemiological and 29 experimental studies (2016–2024) examining e-cigarettes and HTPs in relation to cancer, tumour promotion, genotoxicity and mutagenicity (Supplemental Table 4). Most focused on lung and oral cancers, with some insights into bladder cancer. No studies were found for white snus.

### E-cigarettes

Two recent reviews [17,18] summarized research on biomarkers of exposure. E-cigarette users had elevated urinary levels of carcinogens, including volatile organic compounds, metals (e.g. cadmium) and polycyclic aromatic hydrocarbons. For many other biomarkers such as tobacco-specific nitrosamines, the data were conflicting, likely due to differences in product types and study designs. However, links between these biomarkers and actual cancer risk remain unclear.

Epidemiological data on e-cigarettes and lung cancer remain sparse due to their recent introduction. Bittoni et al. conducted a case-control study (nearly 5000 cases, 27,000 controls) and found that dual use of e-cigarettes and cigarettes increased lung cancer risk fourfold compared with exclusive cigarette smoking [19]. This underscores the potential for interactive effects following dual use.

Animal studies support these findings (Supplemental Table 4). Tang et al. showed that long-term exposure to e-cigarette smoke led to lung adenocarcinoma and bladder urothelial hyperplasia in mice [20]. Mice exposed to e-cigarettes had increased deoxyribonucleic acid (DNA) adducts (indicative of genotoxicity) in the lung, urinary bladder and heart, and decreased DNA repair activity in the lung, underscoring mechanisms like oxidative DNA damage and impaired repair that may drive cancer development (Supplemental Table 4). Further experimental studies indicate that e-cigarettes promote tumour progression in mice primarily by reduced activation of apoptosis in metastatic cancer cells (Supplemental Table 4).

Genotoxicity studies report DNA damage, oxidative stress and chromosomal aberrations after e-cigarette exposure in vivo and in vitro, with nicotine playing a key role (Supplemental Table 4). For example, nicotine-containing e-cigarettes caused significant oxidative DNA damage and increased γH2AX, a marker of DNA strand breaks, in mice. Similar results were seen in human lung and oral cells exposed in vitro. Not all studies agreed: some in-vitro tests found no significant DNA damage or mutations, suggesting effects depend on product type, nicotine concentration and exposure regimens. Nonetheless, evidence indicates the potential for e-cigarettes to induce DNA damage and tumour-promoting changes, especially in lung and oral cells.

The oral cavity is the first site of exposure to e-cigarette aerosols. Epidemiological studies have generally found no significant association between e-cigarette use and precancerous oral lesions [21]. However, biomarkers such as chromosomal aberrations and DNA damage were elevated in the oral mucosa of users [22,23]. Epigenetic studies have reported smoking-like effects, including hypermethylation of cancer-related genes in buccal epithelial cells, indicating potential carcinogenic risks [23,24]. Experimental studies reinforce these concerns. In-vitro work shows that e-cigarette exposure causes chromosomal aberrations and DNA damage in human oral cells, sometimes in a dose-dependent manner and independent of nicotine content (Supplemental Table 4).

### Heated tobacco products

Epidemiological data on HTPs are limited (Supplemental Table 4). However, a number of small observational studies reported increased chromosomal abnormalities in buccal cells and the presence of urinary biomarkers of carcinogen exposure. Mechanistic studies indicate that HTP smoke extracts trigger epithelial-to-mesenchymal transition in lung adenocarcinoma cells, with increased gene expression (Supplemental Table 4). In human bronchial epithelial cells exposed at the air–liquid interface, HTP, but not e-cigarettes, caused higher chromosomal aberrations and oxidative DNA strand breaks at equivalent puff counts. Similar damage was observed in rat lungs and models of human bronchial and alveolar tissue, consistently implicating oxidative stress as a key mechanism.

### Conclusion

Current evidence suggests that both e-cigarettes and HTPs may promote carcinogenesis through DNA damage, oxidative stress and epigenetic changes. Animal studies demonstrate that e-cigarettes can induce lung cancer and bladder lesions, while epidemiological data link dual e-cigarette/cigarette use to increased lung cancer risk. Both products cause DNA damage and tumourigenic changes in cell models. However, since these products are relatively new and cancer develops over long periods, large-scale, long-term studies are critical, to fully assess their cancer risk.

## Cardiovascular diseases

Several systematic and umbrella reviews on e-cigarettes and CVD were identified (Supplemental Table 5) while there were no studies found on HTPs or white snus and CVD.

### E-cigarettes

We identified four systematic reviews/meta-analyses [9,25-27], two umbrella reviews [28,29] and one review [30] relevant to the scope. Two systematic reviews/meta-analyses [9,25] and two umbrella reviews [28,29] synthesized epidemiological studies on e-cigarettes and clinically manifested CVD. One review included seven US-based cross-sectional studies (*n* = 864,888) examining exclusive e-cigarette use in relation to myocardial infarction, stroke and other CVD outcomes and reported a non-significant increase in CVD risk (OR 1.24, 95% CI 0.93–1.67) [25]. Another included 11 studies (*n* = 1,112,152), four of which were longitudinal [9]. The pooled cross-sectional estimate indicated an elevated CVD risk in e-cigarette users (OR 1.24, 95% CI 1.05–1.46). A separate analysis suggested a potential increase in stroke risk (OR 1.32, 95% CI 0.99–1.76). The four longitudinal studies, based on the US PATH and Kaiser Permanente cohorts, showed pooled estimates of OR 1.02 (95% CI 0.91–1.14) for CVD and OR 1.70 (95% CI 1.00–2.80) for stroke. Most available data are derived from cross-sectional studies, limiting causal inference. Additional limitations include recall bias, differences in outcome definitions and limited longitudinal evidence.

The two umbrella reviews concluded that available evidence was insufficient to establish a causal link between e-cigarette use and clinically manifest CVD [28,29]. No studies were identified on atrial fibrillation, abdominal aortic aneurysm or peripheral artery disease.

Evidence on subclinical cardiovascular outcomes is summarized in three systematic reviews [9,26,27] and two umbrella reviews [28,29]. Siddiqi et al. conducted a meta-analysis of 27 randomized crossover studies (*n* = 863) assessing acute exposure to nicotine-containing e-cigarettes [27]. Acute use was associated with increased heart rate, blood pressure, pulse–wave velocity, augmentation index (heart rate adjusted), soluble CD40 ligand and P-selectin and decreased flow-mediated dilation. No significant effects were seen with nicotine-free e-cigarettes, except for a small decrease in mean arterial pressure (−0.89 mmHg, 95% CI −1.26 to −0.51), suggesting nicotine as the primary driver of these changes. Lee et al. focused on the association between e-cigarette use and flow-mediated dilation, including four cross-sectional studies (*n* = 769) [13]. Pooled results showed a non-significant reduction in flow-mediated dilation among exclusive users versus never users (mean difference: −1.47, 95% CI −3.96 to 1.02). Glantz et al. also evaluated cardiometabolic outcomes using 11 studies (mostly cross sectional, plus one longitudinal) from the US, Sweden and Korea [9]. They found a pooled OR of 1.25 (95% CI 1.18–1.33) for composite cardiometabolic disorders, including prediabetes, hypertension, metabolic syndrome and waist circumference. In the umbrella review by Travis et al., acute use of e-cigarettes was associated with elevated heart rate and blood pressure, endothelial dysfunction, increased arterial stiffness and oxidative stress markers [29]. However, evidence regarding long-term cardiovascular effects (e.g. blood pressure, heart-rate variability, cardiac geometry) was deemed insufficient. Banks et al. also concluded that data were lacking for outcomes such as carotid intima-media thickness and coronary artery calcification, especially among never-smokers [28].

The cardiovascular effects of e-cigarettes may be mediated through various mechanisms. These include exposure to fine and ultrafine particles, nicotine, reactive oxidants (e.g. acrolein, formaldehyde, acetaldehyde), heavy metals and flavourings. Proposed mechanisms involve sympathetic nervous system activation, oxidative stress, endothelial dysfunction and increased platelet activation [31]. A review of in-vitro and in-vivo studies reported consistent findings of increased reactive oxygen species’ production, reduced antioxidant levels and impaired endothelial function in human cell models [29]. In animal models, e-cigarette exposure was associated with vascular inflammation, angiogenesis, oxidative stress and increased atherosclerotic plaque formation. Kennedy et al. reviewed eight in-vitro studies using endothelial cells from various origins [30]. E-cigarette exposure consistently increased oxidative stress and impaired endothelial and platelet function. Five animal studies in the same review demonstrated variable effects on cardiac remodelling but consistently showed vascular dysfunction, including mitochondrial damage, lipid accumulation, altered gene expression and markers of inflammation and apoptosis. Long-term exposure was associated with increased arterial stiffness and a shift in vascular tone from vasodilation to vasoconstriction. Platelet studies showed increased aggregation, integrin activation, secretion, prostacyclin resistance and shortened bleeding and occlusion times.

### Conclusions

Findings from epidemiological, in-vitro and in-vivo studies suggest adverse cardiovascular effects associated with e-cigarette use. The evidence so far is mainly from cross-sectional data, which limits drawing firm conclusions. There is a clear need for high-quality prospective studies, particularly in never-smokers, to assess long-term cardiovascular risks. No data are currently available on HTPs or white snus. Given the high nicotine levels in these products, potential cardiovascular risks should be considered.

## Diabetes

We identified eight epidemiological (seven on e-cigarettes, one on HTPs) and six animal studies (five on e-cigarettes, one on oral nicotine) investigating the new nicotine products in relation to diabetes-related outcomes (Supplemental Table 6).

### E-cigarettes

There were seven studies on e-cigarettes and diabetes, prediabetes, insulin resistance or the metabolic syndrome published between 2019 and 2023 [32-38] (Supplemental Table 6). All compared e-cigarette users with non-users, adjusting for confounders such as other tobacco product use, body mass index, socioeconomic status and physical activity. While none specified diabetes type, their focus on adults implies they mostly assessed type 2 diabetes or prediabetes. Four studies examined the prevalence of diabetes or prediabetes among e-cigarette users versus non-users. These included two US studies using data from the National Health and Nutrition Examination Survey (NHANES) [32] and the Behavioural Risk Factor Surveillance System [35] and two Korean studies using Korea National Health and Nutrition Examination Survey (KNHANES) [36] and Korean Community Health Surveys [34]. All reported higher odds of diabetes/prediabetes among e-cigarette users. We pooled the results using the inverse variance method. This resulted in an OR of 1.18 (95% CI 1.06–1.31) for current users versus non-users and OR 1.15 (95% CI 1.02–1.30) for former users. In addition, data from NHANES 2015–2018 showed that ever e-cigarette users were more insulin resistant than never users [32]. However, an earlier NHANES dataset (2013–2016) found no association between e-cigarette use and glucose levels after an oral glucose tolerance test [37]. Regarding metabolic syndrome, NHANES data indicated that current e-cigarette users had a 30% higher prevalence (95% CI 1.13–1.50) compared with never users [33]. KNHANES data showed a similar, though statistically nonsignificant, association (OR 1.27, 95% CI: 0.96–1.70) [36]. One study examined plasma metabolites, finding that e-cigarette use was linked to altered metabolites in the tricarboxylic acid cycle, suggesting disruptions in energy metabolism and potential effects on glucose regulation and oxidative stress [38].

Five animal studies explored the impact of e-cigarette vapour exposure on glucose tolerance and insulin resistance in mice (Supplemental Table 6). One study showed increased insulin resistance after nicotine-containing vapour exposure, while others reported no effects on insulin sensitivity or glucose tolerance. Similar null results were seen in a study exploring effects of nicotine-free e-cigarette exposure. One study reported oxidative stress and disruptions in energy metabolism, highlighting possible mechanisms through which e-cigarettes might impair glucose regulation (Supplemental Table 6).

### White snus

No epidemiological or human experimental studies were found on white snus in relation to diabetes. An animal study in obese rats (Supplemental Table 6) found that chronic oral nicotine lowered plasma glucose after insulin loading, suggesting improved insulin resistance, possibly via reduced hepatic glucose output.

### Heated tobacco products

Only one study examined the association between HTPs and diabetes-related outcomes (Supplemental Table 6). This Japanese cross-sectional study found that exclusive HTP users had higher odds of prediabetes (OR 1.36, 95% CI 1.25–1.47) and diabetes (OR 1.68, 95% CI 1.45–1.94) compared with never-smokers. Higher glycosylated haemoglobin and fasting glucose levels were also observed in HTP users. No animal or human experimental studies were found.

### Conclusions

Limited epidemiological evidence indicates an association between e-cigarette use and increased prevalence of type 2 diabetes, prediabetes, insulin resistance and metabolic syndrome. However, all studies were cross sectional and relied on self-reported data, which raises concerns about reverse causation and recall bias. No study addressed dose or duration of use. Animal studies suggest potential metabolic effects of e-cigarettes, but results are mixed. No studies have assessed the effects of white snus on diabetes risk, and only one study has examined HTPs. Given that nicotine impairs insulin sensitivity and glucose regulation [3], and that newer nicotine products like white snus deliver nicotine at levels comparable with or higher than cigarettes, it is plausible that these products may also increase the risk of diabetes. However, this hypothesis remains to be confirmed.

## Lung diseases

Emerging evidence suggests adverse respiratory effects of e-cigarette use. We reviewed two recent meta-analyses on this topic [9,39]. No relevant epidemiological studies or meta-analyses were found for HTPs. Mechanistic reviews and selected experimental studies using realistic aerosol exposure models are also discussed. White snus was excluded, as it is not inhaled and thus not relevant to lung outcomes.

### E-cigarettes

One of the most significant acute health concerns linked to e-cigarette use has been the outbreak of e-cigarette or vaping-product-use-associated lung injury (EVALI). By February 2020, over 2800 cases and 68 deaths were reported in the US [40]. A study involving 53 patients (median age 19) revealed symptoms such as respiratory distress, chest pain and cough [41]. Radiological and histopathological findings included bilateral infiltrates, ground-glass opacities, lipid-laden macrophages and interstitial inflammation [41]. Laboratory data showed that vitamin E acetate, an additive in some tetrahydrocannabinol-containing e-cigarette products, is strongly linked to the EVALI outbreak.

There is also evidence linking e-cigarette use to chronic bronchitis. Several aerosol constituents in e-cigarettes damage airway linings and interfere with mucociliary clearance [42]. A Swedish study among young adults observed increased symptoms like coughing and mucus production among e-cigarette users, particularly among dual users [42]. A systematic review including eight studies (seven short term, one long term; 273 participants) reported that while airway resistance increased with e-cigarette use, traditional lung function parameters (e.g. forced expiratory volume in 1 s, forced vital capacity) were unaffected [39]. However, the limited size and follow-up time of these studies indicate that larger studies with longer follow-up time are required to determine the effects of e-cigarettes on lung function from a longer perspective.

A meta-analysis of 94 cross-sectional and 30 longitudinal studies found that compared with non-users, both e-cigarette (OR 1.46, 95% CI 1.31–1.61) and dual use (OR 3.29, 95% CI 1.97–5.51) were associated with increased COPD prevalence [9]. Compared with smokers, the OR for COPD was lower for e-cigarette users (OR 0.53, 95% CI 0.38–0.74) but significantly higher in dual users (OR 1.41, 95% CI 1.12–1.64). The relatively lower risk among e-cigarette users may be attributed to their younger age, as COPD typically manifests after age 40 and longer exposure.

In-vitro studies using bronchial and alveolar cell models exposed to fruit-flavoured e-cigarette aerosols demonstrated inflammatory responses, oxidative damage, impaired antimicrobial defence and epigenetic changes [43]. Variables such as flavour type, nicotine concentration, power settings and lung region influenced toxicity [43]. Another study showed that flavoured aerosol exposure, regardless of nicotine content, altered macrophage phenotype, induced pro-inflammatory states, disrupted lipid balance and impaired phagocytosis [44].

Many flavouring chemicals in e-liquids, such as diacetyl and 2,3-pentanedione, are associated with serious respiratory conditions. Diacetyl has been linked to bronchiolitis obliterans, or ‘popcorn lung’, a disease characterized by scarring of the small airways and irreversible breathing difficulties [45]. Despite regulatory bans, diacetyl remains present in some flavoured products. In-vitro testing showed that diacetyl causes inflammation, oxidative stress and epithelial injury [46]. Other compounds like cinnamaldehyde reduce ciliary function, impairing the lungs’ ability to clear mucus [45]. Additional agents such as vanillin and benzaldehyde, used in sweet-flavoured e-liquids, have also been shown to increase lung inflammation and oxidative stress [45]. Although these substances are considered safe for ingestion, inhalation poses entirely different risks. Heating these compounds during vaping can produce unpredictable and potentially harmful degradation by-products [43].

### Heated tobacco products

While HTPs heat rather than burn tobacco, they still emit toxic chemicals that can irritate the respiratory tract and impair lung function. In fact, the aerosol from some HTPs contains concentrations of harmful constituents that are 2–137 times higher than those in mainstream smoke from conventional cigarettes [47]. Because HTPs are relatively new, long-term epidemiological data remain sparse. One in-vitro study exposed bronchial and alveolar models to HTP aerosol using a standardized puffing protocol and assessed various endpoints [48]. The findings highlighted oxidative stress, DNA damage, lipid peroxidation and ferroptosis as key toxicological responses. These effects were consistent with those observed from traditional cigarette smoke exposure and varied between bronchial and alveolar tissue regions [48].

### Conclusion

In summary, the available evidence, from population-based studies, mechanistic experiments and chemical analyses, points to considerable respiratory risks associated with both e-cigarettes and HTPs. Users experience more respiratory symptoms, increased airway resistance and higher odds of developing COPD. EVALI remains a serious acute risk, particularly when products are obtained from unregulated sources. Long-term studies are urgently needed, but current data support the conclusion that these products pose significant threats to respiratory health, potentially leading to conditions like EVALI, chronic bronchitis and COPD.

## Pregnancy

We identified 16 epidemiological studies on pregnancy outcomes (13 on e-cigarettes, 2 on HTPs and 1 on both) together with 17 experimental studies (Supplemental Tables 7, 8). No studies on white snus and pregnancy were found.

### E-cigarettes

Fourteen studies assessed e-cigarette use and pregnancy outcomes, with sample sizes ranging from 248 to 190,707 (Supplemental Table 7). Study types included one randomized–controlled trial (RCT), four cohort studies, eight cross-sectional studies and one ecological study. Most were US based, with eight studies using data from the Pregnancy Risk Assessment Monitoring System (PRAMS).

Small-for-gestational age (SGA) was the most frequently assessed outcome. Two studies reported an increased risk of SGA among exclusive e-cigarette users (relative risk (RR) 1.6–2.4) compared with non-users [49,50], while four others found no significant association [51-55]. These conflicting findings likely reflect differences in study design, inclusion criteria and exposure definitions within PRAMS-based analyses.

Preterm birth showed more consistent associations with e-cigarette use [49-51,53], while results were mixed for low birth weight [49,51-53]. In a UK-based RCT of 1140 pregnant women, Hajek et al. found that e-cigarettes were associated with a lower risk of low birth weight compared with nicotine patches (RR 0.65, 95% CI 0.47–0.90) [56]. A cohort study by Lin et al. (*n* = 597) found no differences in high-risk birth or foetal death between e-cigarette users and non-users [57].

Other outcomes include fecundability, with Harlow et al. reporting a non-significant reduction among e-cigarette users (RR 0.85, 95% CI 0.68–1.07) [58]. Galanti et al. found no major differences in oocyte quality in women undergoing intracytoplasmic sperm injection, except for a higher number of germinal vesicles among e-cigarette users compared with conventional smokers [59]. Wen et al. found no link between e-cigarette use and poor gestational weight gain [55].

Seventeen rodent studies examined gestational and/or lactational exposure to e-cigarette aerosols (Supplemental Table 8). Nicotine-containing aerosols were mostly associated with decreased birth weight or lower weight gain from early-life to adulthood. Nicotine-free aerosol exposure mainly increased postnatal weight. Several rodent studies also reported behavioural changes such as increased hyperactivity, reduced anxiety, memory deficits and impaired locomotor and learning performance. Exposure also worsened outcomes after hypoxic–ischaemic brain injury. At the molecular level, gestational exposure disrupted blood–brain barrier integrity, altered gene expression, increased hippocampal superoxide dismutase activity, and affected neuronal differentiation, calcium signalling, microglial function and neuroinflammation.

### Heated tobacco products

Epidemiological evidence on HTPs in pregnancy is limited to three studies (Supplemental Table 7). In an Italian cohort of 642 pregnant women, higher preterm birth rates were found among HTP users (17%) versus non-smokers (4%), although no statistical testing was performed. A Japanese cross-sectional study of 558 postpartum women found non-significant increases in hypertensive disorders (OR 2.78, 95% CI 0.84–9.15) and low birth weight (OR 2.08, 95% CI 0.80–9.15) among HTP ever users. Another study reported no major differences in oocyte quality between HTP and cigarette users. Only one experimental study on HTP exposure during pregnancy was identified (Supplemental Table 8). Prenatal exposure to HTPs in rodents led to abnormal testicular morphology and decreased spermatogenesis in male offspring.

### Conclusion

Current epidemiological evidence suggests potential risks of e-cigarettes during pregnancy, particularly for preterm birth, low birth weight and SGA, though findings remain mixed. Data on stillbirth and infant mortality are sparse. Mechanistic studies support associations with impaired growth and neurodevelopment, though inconsistencies remain.

For HTPs, data are extremely limited, and no studies exist on white snus in pregnancy. The adverse effects of traditional snus use during pregnancy are well documented and include stillbirth, extremely and moderately premature birth, SGA, low birth weight, oral cleft malformations and neonatal apnoea. Given the shared nicotine content of these products, similar risks may be expected from white snus, warranting urgent research.

## Conclusions

This review provides a comprehensive assessment of the current evidence on health risks associated with emerging nicotine and tobacco products, e-cigarettes, HTPs and white snus, focusing on allergic, cardiovascular and respiratory diseases, cancer, diabetes and adverse pregnancy outcomes (summarized in Figure 1). As patterns of nicotine consumption shift globally, with conventional cigarette use declining and novel products gaining popularity, especially among adolescents and young adults, understanding the health consequences of these products is critical. The increased use in women of reproductive age is particularly alarming.

**Figure 1.**
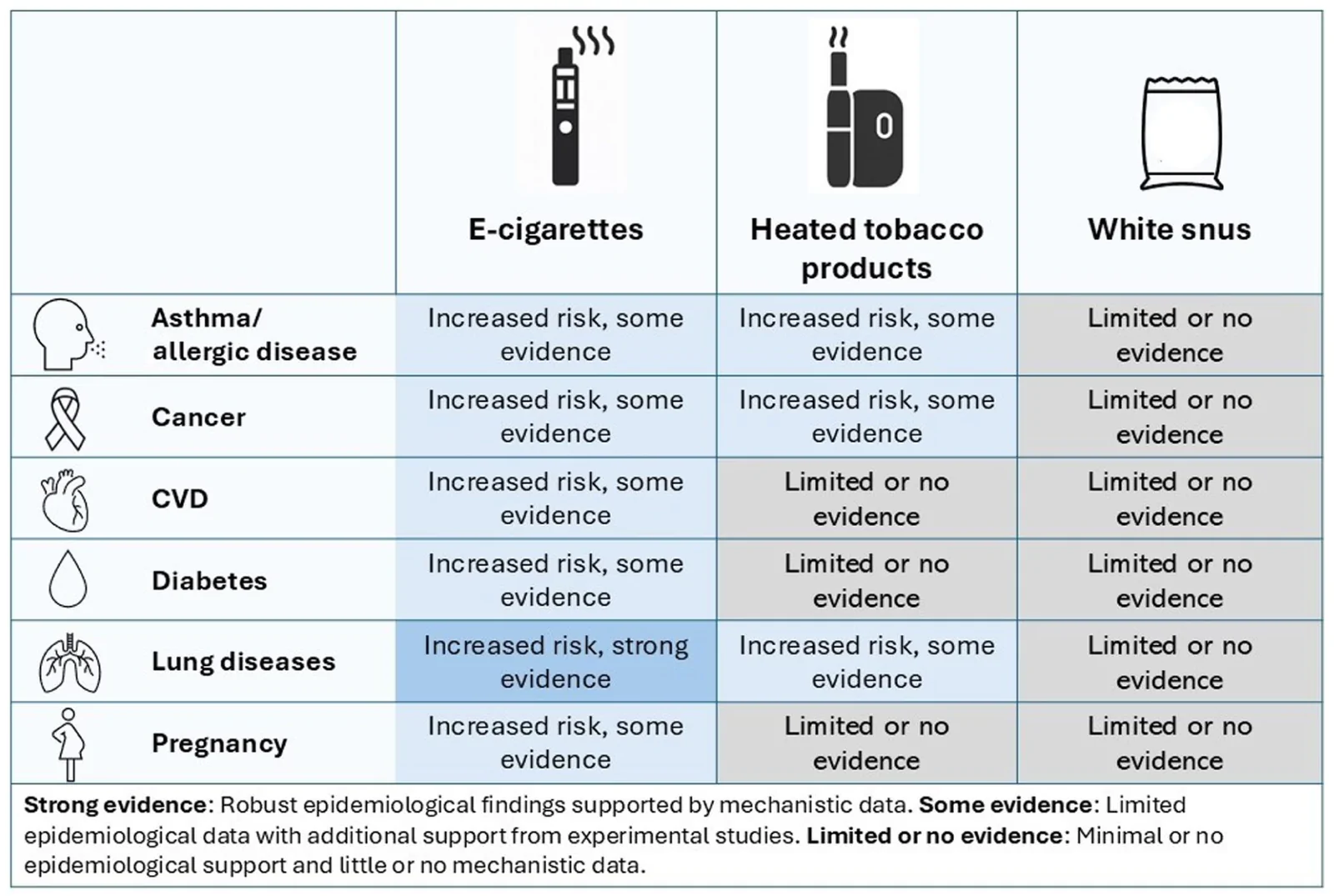
Summary of current evidence on e-cigarettes, HTPs and white snus and asthma/allergy, cancer, cardiovascular disease, cancer, diabetes, lung diseases and pregnancy outcomes. CVD: cardiovascular disease; HTP: heated tobacco products.

Our review shows that the use of e-cigarettes is associated with increased risks of asthma, COPD and other respiratory conditions (Table I). These findings are supported by mechanistic data from experimental studies demonstrating inflammatory and structural effects on the respiratory system. There is also limited, yet concerning, evidence linking e-cigarettes to CVD, type 2 diabetes, cancer and adverse pregnancy outcomes. However, most of the epidemiological studies are cross sectional, limiting causal inference due to potential reverse causation and residual confounding, particularly by concurrent or past cigarette smoking.

**Table I.** Summary of literature review: Epidemiological studies (mainly cross sectional) investigating asthma/allergic disease, cancer, CVD, diabetes, COPD or pregnancy outcomes in relation to use of e-cigarettes, white snus and heated tobacco products (use versus no use or never use).

Outcome	Studies	Outcome	Pooled estimate (OR/RR 95% CI)
E-cigarettes			
Asthma/Allergic disease	Glantz et al. [9] (meta-analysis of 41 studies)	Asthma/wheeze	1.24 (1.19–1.30)
	Lee et al. [13], Smith et al. [15], Youn et al. [16]	Atopic dermatitis	1.46 (1.26–1.69)
Cancer	No studies identified		
CVD	Glantz et al. [9] (meta-analysis of 11 studies for CVD, 5 studies for stroke)	CVD and Stroke	CVD: 1.24 (1.05–1.46) Stroke: 1.32 (0.98–1.76)
Diabetes	Cai and Bidulescu [32], Jeong and Kim [34], Zhang et al. [35], Kim et al. [36]	Prediabetes/diabetes	1.18 (1.06–1.31)
COPD	Glantz et al. [9] (meta-analysis of 19 studies)	COPD	1.46 (1.31–1.61)
Pregnancy outcomes	Kim et al. [49], Wang et al. [50], Wen et al. [55], Ammar et al. [53], Cardenas et al. [60]	SGA (<10 percentile)	1.37 (1.08–1.74)
	Kim et al. [49], Wang et al. [50], Regan et al. [51], Ammar et al. [53]	Preterm birth (<37 weeks)	1.47 (1.22–1.78)
Heated tobacco products			
Asthma/allergic disease	Noguchi et al. (2023)^a^, Nakama and Tabuchi (2021)^a^, Seo et al. (2023)^a^, Lee et al. (2019)^a^	Asthma	1.67 (1.06–2.68)
	Seo et al. (2023)^a^, Lee et al. (2019)^a^	Allergic rhinitis	1.24 (1.10–1.41)
Cancer	No studies identified		
CVD	No studies identified		
Diabetes	Hu et al. (2022)^b^	Prediabetes and diabetes	Prediabetes 1.36 (1.25–1.47) Diabetes 1.68 (1.45–1.94)
COPD	No studies identified		
Pregnancy outcomes	Zaitsu et al. (2021)^c^	Low birth weight	2.08 (0.80–5.40)
White snus			
No epidemiological studies on any of the outcomes identified			

Pooled estimates were obtained from the largest available meta-analysis for outcomes related to e-cigarettes and asthma, cardiovascular disease (CVD) and chronic obstructive pulmonary disease (COPD) [9]. For outcomes without existing meta-analyses, we pooled effect estimates from individual studies using the inverse variance method, provided at least two studies reported relative effect measures. This approach was applied to the association between e-cigarettes and diabetes, small-for-gestational age (SGA), preterm birth and the association between heated tobacco products (HTPs) in relation to asthma and allergic rhinitis. When only one relevant study was available, its estimate is presented (HTPs and diabetes or low birth weight).

a Supplemental Table 3.

b Supplemental Table 6.

c Supplemental Table 7.

CI: confidence interval; OR: odds ratio; RR: relative risk.

In contrast, evidence regarding HTPs is more limited. A small number of epidemiological studies suggest associations with asthma and COPD (Table I), findings that are biologically plausible given the toxic compounds generated during product use. Experimental studies support respiratory toxicity of HTPs, but data on other health outcomes, such as CVD or diabetes, remain sparse. Methodological limitations, such as short follow up, inadequate comparison groups (e.g. conventional smokers) and self-reported exposure, further weaken the strength of evidence.

For white snus, the knowledge base is even more deficient. To date, there are virtually no studies evaluating its long-term health impacts. Given its high nicotine content and similarities to traditional Swedish snus, it is reasonable to hypothesize that white snus may carry similar risks, particularly with respect to type 2 diabetes and adverse pregnancy outcomes. This concern is amplified by its rising popularity among adolescents and young women in Sweden, a trend that raises public health alarms, particularly due to the potential for prenatal exposure and its consequences on foetal and child development.

The marketing strategies for these new products frequently target individuals who are not current smokers, raising the risk of nicotine initiation and subsequent addiction. Thus, here is concern that these products may serve as gateways to traditional tobacco use, contradicting industry claims that they aid in smoking cessation or harm reduction. From a public health standpoint, no form of nicotine dependence should be considered acceptable or benign.

Despite limitations in the current evidence base, most notably, the lack of long-term prospective data and methodological challenges such as insufficient adjustment for confounders, there is already sufficient concern to warrant preventive measures. Public health policies should not wait for definitive proof of harm, particularly given the addictive nature of nicotine and the known latency of diseases such as cancer and CVD.

Results from available epidemiological studies, including effect estimates, are presented in Table I. To strengthen the evidence base, future research should prioritize longitudinal epidemiological studies that can capture long-term exposure and outcomes, particularly for newer products like white snus. These studies should incorporate detailed exposure assessment, consider dual or poly-product use and account for sociodemographic factors that may influence both product use and health risks. In parallel, experimental studies are needed to elucidate biological mechanisms, identify early biomarkers of disease and evaluate the toxicity of specific product constituents such as flavouring agents and carrier substances.

Given the increasing use of these products among women of childbearing age, targeted research is urgently needed to assess effects on fertility, pregnancy outcomes and offspring health. Innovative communication strategies and behavioural interventions must also be developed and tested to address this emerging risk group and prevent intergenerational harm.

The environmental impact of new nicotine and tobacco products is another emerging concern. Nicotine is usually derived from tobacco, thus sharing the environmental burden of cultivation such as deforestation, pesticide use and high water demand [61]. In addition, disposable e-cigarettes, HTPs and white snus generate substantial plastic, metal and battery waste, much of which is improperly discarded and rarely recycled. Such waste contributes to pollution and may indirectly affect public health. Future research should assess the scale of these problems, and regulations should promote sustainable product design, waste management and producer responsibility.

In summary, although conclusive evidence is still developing, the existing literature already signals a range of potential health risks associated with e-cigarettes, HTPs and white snus (Figure 1). Given the addictive potential and the plausible biological pathways linking these products to serious health outcomes, proactive public health action is both warranted and necessary. Ongoing surveillance, high-quality research, and effective prevention strategies will be essential to mitigate the future health burden of these increasingly prevalent nicotine products.

## Supplemental Material

sj-docx-1-sjp-10.1177_14034948251391690 – Supplemental material for New nicotine and tobacco products and risk of major diseases: A review of the scientific evidence Supplemental material, sj-docx-1-sjp-10.1177_14034948251391690 for New nicotine and tobacco products and risk of major diseases: A review of the scientific evidence by Sofia Carlsson, Miranda Beck, Anna Bergström, Kristian Dreij, Koustav Ganguly, Maria Kippler, Federica Laguzzi, Karin Leander, Donghau Lu, Lena Palmberg, Swapna Upadhyay, Anna Zettergren and Göran Pershagen in Scandinavian Journal of Public Health
