# Supplementary material for: New nicotine and tobacco products and risk of major diseases: A review of the scientific evidence
Source: Scand J Public Health. 2025 Nov 12;54(8):942–53. doi: 10.1177/14034948251391690 (PMC13710727; doi:10.1177/14034948251391690)
Supplement: Supplementary file 1 [file sj-docx-1-sjp-10.1177_14034948251391690.docx]

## Supplemental to “Health effects of new nicotine and tobacco products-A review of the scientific evidence”

Carlsson S, Beck M, Bergström A, Dreij K, Ganguly K, Leander K, Laguzzi F, Lu D, Kippler M, Palmberg L, Upadhyay S, Zettergren A, Pershagen G.

Institute of Environmental Medicine, Karolinska Institutet, Stockholm, Sweden.

**Supplemental Table 1. Summary of literature review: Epidemiological studies on e-cigarette use and allergic disease**

| Study | Design | Population | Exposure & Outcome | Adjusted for | Key Findings (OR/HR, 95% CI) |
| --- | --- | --- | --- | --- | --- |
| Chand & Hosseinzadeh, 2022 | Systematic Review and Meta-Analysis (13 studies) | Adolescents & adults | E-cig use → Asthma diagnosis | 10 studies considered of "good" and 4 studies of "fair" quality according to AXIS criteria | Current: 1.36 (1.21–1.52); Ever: 1.24 (1.13–1.36) |
| Glantz et al., 2024 | Systematic Review and Meta-Analysis (41 studies) | All ages | Exclusive e-cig use → Asthma/wheeze | Low risk of bias according to ROBINS-E criteria. | 1.24 (1.19–1.30) |
| Wills et al., 2021 | Systematic Review and Meta-Analysis (15 studies) | Adolescents & adults | E-cig use → Asthma diagnosis/symptoms | NA | 1.39 (1.28–1.51) |
| Alqahtani et al., 2023 | Systematic Review and Meta-Analysis (6 studies) | Adults | Exclusive e-cig use → Wheeze | 4 studies considered to have low, and 2 studies unclear risk of bias according to NIH Study Quality Assessment Tools. | Pooled prevalence: 19% (12–30%) |
| Xian & Chen, 2021 | Systematic Review and Meta-Analysis (11 studies) | Adolescents & adults (South Korea, USA) | E-cig use & Asthma diagnosis | 6 studies high and 5 studies moderate risk of bias (AHQR) | Current: 1.30 (1.17, 1.45), Former: 1.22 (1.08, 1.39) |
| Li et al., 2022 | Systematic Review and Meta-Analysis (10 studies) | Adolescents | E-cig use → Asthma diagnosis | Moderate risk of bias (AHQR) | Ever: 1.31 (1.22, 1.42); Current: 1.36 (1.26, 1.48); Former: 1.20 (1.12, 1.28) |
| Delmas et al., 2024 | Cross-sectional | Adults (n=121,186, France) | E-cig use → Asthma symptom score | Multiple including smoking, BMI | Current: 1.34 (1.28, 1.41); Former: 1.39 (1.33, 1.45); Never smokers: 1.40 (1.14, 1.72) |
| Dirisanala et al., 2023 | Cross-sectional | Adults (USA, NHANES) | Ever e-cig use → Asthma | Demographics, comorbidities | 1.47 (1.21–1.78) |
| Hedman et al, 2024 | Cross-sectional | Age 16–69, Sweden, n=17,325 | E-cig use & respiratory symptoms | Sex, age, SES, baseline symptom count | E-cig among never smokers: 0.48 (0.10, 2.29) |
| Jackson et al, 2020 | Cross-sectional | Healthy adults, USA, 2016–2019, n=46 | E-cig use (cotinine validated) & IgE/IgG levels | None | Higher IgE in e-cig users vs non-smokers; no difference in IgG |
| Kioi & Tabuchi, 2018 | Cross-sectional | Adults aged 40–69, Japan, 2015, n=4,432 | E-cig use & asthma/atopic dermatitis | IPW by demographics incl. education, housing tenure | Asthma: 13% (ever use) vs 3.2% / 1.2%; Dermatitis: 13% vs 3.5% / 1.2% (current) |
| Lee et al, 2019 | Cross-sectional | Students aged 12–18, South Korea, 2018, n=58,336 | Ever e-cig use & diagnosis of asthma, allergic rhinitis, atopic dermatitis | Age, sex, obesity, residence, SES, physical activity | Asthma: 1.23 (1.00–1.52); Rhinitis: 1.08 (1.00–1.18); Dermatitis: 1.32 (1.18–1.49); Exclusive use → Dermatitis: 1.34 (1.00–1.80) |
| Rha et al, 2022 | Cross-sectional | Adults over 19, KNHANES, South Korea (n=38,413) | Current and former e-cig use → Allergic rhinitis diagnosis | Age, BMI, sex, income, education, smoking, alcohol, HTN, DM, asthma, dermatitis, TB | Current: 1.38 (1.15–1.66); Former: 1.06 (0.94–1.20) |
| Roh et al, 2023 | Cross-sectional | High school students 13–17, YRBSS, USA (TX: n=3042; US: n=32,885) | Ever e-cig use (never smokers) → Ever asthma diagnosis | Age, sex, race/ethnicity, BMI, combustible & other substance use, depression | TX: 1.32 (1.06–1.66); US: 1.18 (1.02–1.37) |
| Smith et al, 2023 | Cross-sectional | Adults >18, NHIS, USA (n=28,563) | Ever e-cig use → Atopic dermatitis | Age, education, race, income, sex, diabetes, smoking status, asthma, BMI | All: 1.35 (1.16–1.58); Never smokers: 1.61 (1.28–2.02) |
| Sompa et al, 2022 | Cross-sectional | Young adults 22–25, BAMSE cohort, Sweden (n=3055) | Current exclusive e-cig use → Breathing difficulties or wheeze | Sex, education, occupation, waterpipe, second-hand smoke, past tobacco use | 1.2 (0.3–3.8) |
| Yao et al, 2024 | Prospective | Adolescents 12–17, PATH, USA (n=11,748) | Current e-cig use → Wheeze, new asthma, asthma interference | Cannabis use, age, sex, race/ethnicity, income, home rules, smoking in home, baseline wheeze | Wheeze: 1.03 (0.69–1.51); Asthma: 0.37 (0.07–1.87); Interference: 1.39 (0.63–3.09) |
| Youn et al, 2024 | Cross-sectional | Households with children <18, NHIS, USA (n=35,479) | Parental e-cig use → Child atopic dermatitis | Parental smoking, asthma, rhinitis, allergy history, demographics, region | All: 1.24 (1.08–1.42); Non-smokers: 1.37 (1.05–1.78) |
| Pérez et al., 2024 | Prospective (PATH) | Adults & adolescents (USA) | Current e-cig use → Asthma onset | Demographics, substance use | Adults: HR 3.52 (1.24–10.02); Youth: NS |
| Qeadan et al., 2023 | Prospective (PATH) | Adults (USA) | Exclusive e-cig use → Asthma incidence | Demographics, health status | 1.12 (0.99–1.27) |

NS, Not significant; E-cig, Electronic cigarettes; NA, Not applicable; OR, odds ratio; 95% CI, 95% confidence interval; ROBINS-E, Risk Of Bias In Non-randomized Studies – of Exposure; NIH, National Institutes of Health; AHQR, Agency of Healthcare Research and Quality; BMI, body mass index; NHANES, National Health and Nutrition Examination Survey; OLIN, Obstruktiv Lungsjukdom I Norrbotten; WSAS, West Sweden Asthma Study; IgE, Immunoglobin E; IgG, Immunoglobin G; KYRBWS, Korea Youth Risk Behavior Web-based Survey; COPD, chronic obstructive pulmonary disease; PATH, Population Assessment of Tobacco and Health; KNHANES, Korea National Health and Nutrition Examination Survey; YRBSS, Youth Risk Behavior Surveillance System; BAMSE, Barn Allergi Miljö Stockholm Epidemiologi; NHIS, National Health Interview Survey;

**Supplemental Table 2. Summary of literature review: Experimental studies on e-cigarette exposure and allergic mechanisms**

| Study | Model | Exposure | Main Findings |
| --- | --- | --- | --- |
| Cahill et al., 2022 | In vivo (mouse offspring) | JUUL mint aerosol (gestational) | ↑ Lung inflammation & asthma-related gene expression in offspring |
| Chapman et al, 2019 | In vivo, BALB/c asthma model | BALB/c mice (HDM-challenged); exposed to air or flavoured e-cig aerosols (± nicotine), 2×/day | Nicotine e-cig vapors suppressed airway inflammation but did not affect hyperresponsiveness. Nicotine-free flavored vapors had varied effects on allergic airway disease |
| Gahring et al, 2020 | In vivo, unspecified mouse strain | Nicotine aerosol (40 mg/mL) or nicotine in water; HDM challenge; exposure 2×/day for 8 weeks | Nicotine aerosols reduced eosinophil recruitment via α7 nicotinic receptor, suggesting modulation of allergic airway response |
| Gellatly et al., 2020 | In vitro (human airway cells) | Nicotine-free vs. nicotine vapor | Nicotine-free vapor ↑ IL-6 and MUC5AC; nicotine vapor had no effect |
| Ghosh et al., 2019 | In vitro (human BAL samples) | Nicotine vapor exposure | ↑ MMP-2/9, neutrophil elastase, inflammatory markers |
| Kang and Valerio, 2020 | In silico | Alkenylbenzene and aldehyde flavorants from e-liquids; DNA adduct prediction via modelling tools | High concordance with predicted skin sensitization for both chemical classes |
| Larcombe et al., 2017 | In vivo (mice, 8 weeks) | PG/VG aerosol types (± nicotine) | Glycerine-based aerosols → airway hyperresponsiveness regardless of nicotine content |
| Lee et al., 2024 | In vivo (mouse asthma model) | E-cig vapor + OVA challenge | ↑ Th2 cytokines, mucus production, airway hyperresponsiveness |
| Muthumalage & Rahman, 2023 | In vivo (C57/BL67, BALB/cJ) | Flavoured aerosols ± nicotine (3 days) | Strain-, flavour-, and nicotine-dependent immune and cytokine responses |
| Noël et al., 2023 | In vivo (mouse offspring) | Vanilla-flavoured e-cig vapor during gestation | ↑ Asthma-like features and sex-specific gene dysregulation in male offspring |
| Song et al., 2023 | In vivo (mice, 2-week exposure) | PG/VG ± nicotine, 4 h/day | ↑ Inflammation, mitochondrial damage, sex-specific airway remodelling effects |
| Song et al., 2023 | In vivo (mice, 3-month exposure) | PG/VG ± nicotine, 4 h/day | Nicotine aerosols ↑ inflammation and altered lung gene expression in asthmatic mice |
| Kang & Valerio, 2020 | In silico | Flavouring agents: alkylbenzenes, aldehydes | Strong DNA-adduct formation potential; predicted skin sensitization |
| Riedel et al., 2018 | Human sputum samples | Healthy adults: e-cig users vs. smokers vs. never-smokers | E-cig users had altered mucus profiles (neutrophil degranulation, mucin expression), typical of asthma/COPD |
| Taha et al., 2020 | In vivo (BALB/c mice, OVA model) | E-cig vapor ± OVA, 1 h/day for 4 weeks | ↑ Airway inflammation and remodelling in both sensitized and control mice; more pronounced in asthmatic group |

NET, Neutrophil extracellular traps; HDM, house dust mite; VG, vegetable glycerine; PG, propylene glycol; BALF, bronchoalveolar lavage fluid; OVA, ovalbumin; Th2, T-helper 2 cells; IL-6, interleukin 6.

**Supplemental Table 3. Summary of literature review: Epidemiological Studies on Heated Tobacco Products and Allergic Outcomes**

| Study | Design | Population | Exposure & Outcome | Adjusted for | Key Findings (OR/HR, 95% CI) |
| --- | --- | --- | --- | --- | --- |
| Lee et al, 2019 | Cross-sectional | Students 12–18, Korea, n=58,336 | Ever HTP use & asthma, rhinitis, dermatitis | Age, sex, obesity, SES, activity | Asthma: 1.78 (1.37–2.32); Rhinitis: 1.21 (1.06–1.38); Dermatitis: 1.58 (1.34–1.87); Exclusive HTP use → asthma: 3.59 (1.47–8.78) |
| Kioi & Tabuchi, 2018 | Cross-sectional | Adults 40–69, Japan, n=4,432 | Ever/current HTP use & asthma/dermatitis | IPW: education, housing tenure | Underpowered; no clear conclusions |
| Nakama & Tabuchi, 2021 | Cross-sectional | Adults 15–73, Japan, n=9,008 | Current HTP use & asthma | Age, sex, smoking, income, education, alcohol | Asthma: 3.97 (1.73–9.11) |
| Noguchi et al, 2023 | Cross-sectional | Adults >40, Japan | Current HTP use & asthma | Sex, age, income, education, alcohol | Asthma: OR 0.61 (0.28–1.33) |
| Seo et al, 2023 | Cross-sectional | Adults >19, Korea, n=18,230 | Exclusive HTP use & rhinitis, asthma, dermatitis | Age, sex, SES, obesity, comorbidities | Rhinitis: 1.60 (1.06–2.42); Asthma: 0.59 (0.15–2.33); Dermatitis: 0.87 (0.48–1.58) |
| Yoshioka et al, 2023 | Cross-sectional | Non-tobacco users 15–80, Japan, n=18,839 | Second-hand HTP aerosol & asthma symptoms | Demographics, past tobacco use, SHS, infections | Asthma symptoms: 1.49 (1.21–1.85) |
| Zaitsu et al, 2023 | Cross-sectional | Mother-infant pairs, Japan, n=5,688 | HTP use pre/during/post pregnancy & child allergic disease | Maternal demographics, smoking, allergy history | Child allergy: 1.98 (1.28–3.05) |

KYRBWS, Korea Youth Risk Behavior Web-based Survey; HTP, heated tobacco product; OR, odds ratio; 95% CI, 95% confidence interval; JASTIS, Japan Society and New Tobacco Internet Survey; COPD, chronic obstructive pulmonary disease; ACO, Asthma and COPD; KNHANES, Korea National Health and Nutrition Examination Survey; JACSIS, Japan COVID-19 and Society Internet Survey.

### References for asthma/allergic disease tables

1. Alqahtani MM, Alenezi FK, Almeshari MA, Alanazi AM, Taleb Z Ben, Ebrahimi Kalan ME, et al. E-cigarette use and respiratory symptoms in adults: A systematic review and meta-analysis. Tob Induc Dis. 2023 Dec;21.
2. Cahill KM, Johnson TK, Perveen Z, Schexnayder M, Xiao R, Heffernan LM, et al. In utero exposures to mint-flavored JUUL aerosol impair lung development and aggravate house dust mite-induced asthma in adult offspring mice. Toxicology. 2022 Jul;477.
3. Chand BR, Hosseinzadeh H. Association between e-cigarette use and asthma: a systematic review and meta-analysis. Journal of Asthma. 2022;59(9):1722–31.
4. Chapman DG, Casey DT, Ather JL, Aliyeva M, Daphtary N, Lahue KG, et al. The Effect of Flavored E-cigarettes on Murine Allergic Airways Disease. Sci Rep. 2019 Dec;9(1).
5. Delmas MC, Pasquereau A, Renuy A, Bénézet L, Ribet C, Zins M, et al. Electronic cigarette use and respiratory symptoms in the French population-based Constances cohort. Respir Med. 2024 Jan;221.
6. Dirisanala S, Laller S, Ganti N, Taj S, Patel N, Singh Arora K, et al. E-cigarette use and prevalence of lung diseases among the U.S. population: a NHANES survey. J Investig Med. 2023 Aug;71(6):613–22.
7. Gahring LC, Myers EJ, Rogers SW. Inhaled aerosolized nicotine suppresses the lung eosinophilic response to house dust mite allergen. Am J Physiol Lung Cell Mol Physiol. 2020 Oct;319(4):L683–92.
8. Gellatly S, Pavelka N, Crue T, Schweitzer KS, Day BJ, Min E, et al. Nicotine-Free e-Cigarette Vapor Exposure Stimulates IL6 and Mucin Production in Human Primary Small Airway Epithelial Cells. J Inflamm Res. 2020 Apr 16;13:175–85.
9. Ghosh A, Coakley RD, Ghio AJ, Muhlebach MS, Esther CR, Alexis NE, et al. Chronic E-cigarette use increases neutrophil elastase and matrix metalloprotease levels in the lung. Am J Respir Crit Care Med. 2019 Dec;200(11):1392–401.
10. Glantz SA, Nguyen N, Oliveira da Silva AL. Population-Based Disease Odds for E-Cigarettes and Dual Use versus Cigarettes. NEJM evidence. 2024 Feb;3(3).
11. Hedman L, Lyytinen G, Backman H, Lundbäck M, Stridsman C, Lindberg A, et al. Electronic cigarette use in relation to changes in smoking status and respiratory symptoms. Tob Induc Dis. 2024;22(January):1–11.
12. Jackson M, Singh KP, Lamb T, McIntosh S, Rahman I. Flavor Preference and Systemic Immunoglobulin Responses in E-Cigarette Users and Waterpipe and Tobacco Smokers: A Pilot Study. Int J Environ Res Public Health. 2020 Jan;17(2):640.
13. Kang JC, Valerio LG. Investigating DNA adduct formation by flavor chemicals and tobacco byproducts in electronic nicotine delivery system (ENDS) using in silico approaches. Toxicol Appl Pharmacol. 2020 Jul;398.
14. Kioi Y, Tabuchi T. Electronic, heat-not-burn, and combustible cigarette use among chronic disease patients in Japan: A cross-sectional study. Tob Induc Dis. 2018 Sep;16(September).
15. Larcombe AN, Janka MA, Mullins BJ, Berry LJ, Bredin A, Franklin PJ. The effects of electronic cigarette aerosol exposure on inflammation and lung function in mice. Am J Physiol Lung Cell Mol Physiol. 2017;313(1):L67–79.
16. Lee A, Lee SY, Lee KS. The Use of Heated Tobacco Products is Associated with Asthma, Allergic Rhinitis, and Atopic Dermatitis in Korean Adolescents. Scientific Reports. 2019 Nov;9(1):1–8.
17. Lee YH, Na HG, Choi YS, Bae CH, Song SY, Kim YD. E-cigarettes exacerbate allergic inflammation via cytokine induction and MUC5AC/5B expression in a murine asthma model. Environ Toxicol Pharmacol. 2024 Apr 1;107.
18. Li X, Zhang Y, Zhang R, Chen F, Shao L, Zhang L. Association Between E-Cigarettes and Asthma in Adolescents: A Systematic Review and Meta-Analysis. Am J Prev Med. 2022 Jun;62(6):953–60.
19. Muthumalage T, Rahman I. Pulmonary immune response regulation, genotoxicity, and metabolic reprogramming by menthol- and tobacco-flavored e-cigarette exposures in mice. Toxicol Sci. 2023 Jun;193(2):146–65.
20. Nakama C, Tabuchi T. Use of heated tobacco products by people with chronic diseases: The 2019 JASTIS study. PLoS One. 2021 Nov;16(11):e0260154.
21. Noël A, Yilmaz S, Farrow T, Schexnayder M, Eickelberg O, Jelesijevic T. Sex-Specific Alterations of the Lung Transcriptome at Birth in Mouse Offspring Prenatally Exposed to Vanilla-Flavored E-Cigarette Aerosols and Enhanced Susceptibility to Asthma. Int J Environ Res Public Health. 2023 Feb;20(4).
22. Noguchi S, Ishimaru T, Fujino Y, Yatera K, Tabuchi T. Association of cigarette smoking with increased use of heated tobacco products in middle-aged and older adults with self-reported chronic obstructive pulmonary disease, asthma, and asthma-COPD overlap in Japan, 2022: the JASTIS study. BMC Pulm Med. 2023 Dec;23(1):1–8.
23. Pérez A, Valencia S, Jani PP, Harrell MB. Use of Electronic Nicotine Delivery Systems and Age of Asthma Onset Among US Adults and Youths. JAMA Netw Open. 2024 May;7(5):e2410740–e2410740.
24. Qeadan F, Nicolson A, Barbeau WA, Azagba S, English K. The association between dual use of electronic nicotine products and illicit drugs with adverse cardiovascular and respiratory outcomes in a longitudinal analysis using the Population Assessment of Tobacco and Health (PATH) survey. Drug and Alcohol Dependence Reports. 2023 Jun;7:100166.
25. Reidel B, Radicioni G, Clapp PW, Ford AA, Abdelwahab S, Rebuli ME, et al. E-cigarette use causes a unique innate immune response in the lung, involving increased neutrophilic activation and altered mucin secretion. Am J Respir Crit Care Med. 2018 Feb 15;197(4):492–501.
26. Rha MS, Cho HJ, Yoon JH, Kim CH. Association between the use of electronic cigarettes and the prevalence of chronic rhinosinusitis and allergic rhinitis: a nationwide cross-sectional study*. Rhinology. 2022;60(1):20–8.
27. Roh T, Uyamasi K, Aggarwal A, Obeng A, Carrillo G. Association between e-cigarette use and asthma among US adolescents: Youth Risk Behavior Surveillance System 2015–2019. Prev Med (Baltim). 2023 Oct 1;175.
28. Seo YG, Paek YJ, Kim JH, Kim JK, Noh HM. Relationship between heated tobacco product use and allergic rhinitis in Korean adults. Tob Induc Dis. 2023;21(November):1–9.
29. Smith B, Engel P, Collier MR, Devjani S, Javadi SS, Maul JT, et al. Association between electronic-cigarette use and atopic dermatitis among United States adults. J Am Acad Dermatol. 2023 Jul;89(1):163–5.
30. Sompa SI, Zettergren A, Ekström S, Upadhyay S, Ganguly K, Georgelis A, et al. Predictors of electronic cigarette use and its association with respiratory health and obesity in young adulthood in Sweden; findings from the population-based birth cohort BAMSE. Environ Res. 2022 May;208.
31. Song MA, Kim JY, Gorr MW, Miller RA, Karpurapu M, Nguyen J, et al. Sex-specific lung inflammation and mitochondrial damage in a model of electronic cigarette exposure in asthma. Am J Physiol Lung Cell Mol Physiol. 2023 Nov;325(5).
32. Song MA, Wold LE, Aslaner DM, Archer KJ, Patel D, Jeon H, et al. Long-Term Impact of Daily E-cigarette Exposure on the Lungs of Asthmatic Mice. Nicotine Tob Res. 2023 Dec;25(12):1904–8.
33. Taha HR, Al-Sawalha NA, Alzoubi KH, Khabour OF. Effect of E-Cigarette aerosol exposure on airway inflammation in a murine model of asthma. Inhal Toxicol. 2020;32(13–14):503–11.
34. Wills TA, Soneji SS, Choi K, Jaspers I, Tam EK. E-cigarette use and respiratory disorders: an integrative review of converging evidence from epidemiological and laboratory studies. Eur Respir J. 2021 Jan;57(1).
35. Xian S, Chen Y. E-cigarette users are associated with asthma disease: A meta-analysis. Clin Respir J. 2021 May;15(5):457–66.
36. Yao T, Lea Watkins S, Sung HY, Wang Y, Gu D, Chen Lyu J, et al. Association between tobacco product use and respiratory health and asthma-related interference with activities among U.S. Adolescents. Prev Med Rep. 2024 May;41.
37. Yoshioka T, Shinozaki T, Hori A, Okawa S, Nakashima K, Tabuchi T. Association between exposure to secondhand aerosol from heated tobacco products and respiratory symptoms among current non-smokers in Japan: a cross-sectional study. BMJ Open. 2023 Mar;13(3):e065322.
38. Youn GM, Sarin KY, Chiou AS, Chen JK, Honari G. Parental E-Cigarette Use and Pediatric Atopic Dermatitis. JAMA Dermatol. 2024 Jul;160(7):780–2.
39. Zaitsu M, Kono K, Hosokawa Y, Miyamoto M, Nanishi K, Okawa S, et al. Maternal heated tobacco product use during pregnancy and allergy in offspring. Allergy. 2023 Apr;78(4):1104–12.60.

**Supplemental table 4. Summary of literature review: Studies investigating the new nicotine products in relation to cancer development, tumour promotion and progression, genotoxicity and mutagenicity.**

| Study | Design | Population/Model | Exposure & Outcome | Adjusted for | Key Findings |
| --- | --- | --- | --- | --- | --- |
| E-CIGARETTES | | | | | |
| Bitton et al. (2024) | Case-control | US adults, n=32,269 | Dual use vs. cig only – Lung cancer | Age, sex, race, smoking, vaping | OR: Dual 58.9; Cig only 13.9 |
| Camila et al. (2023) | Cross-sectional | Colombia, n=64 | E-cig – Genotoxicity (blood) | None | ↑ genotoxicity in vapers |
| Cheng et al. (2022) | Cross-sectional | US, n=40 | E-cig – Genotoxicity (oral DNA adducts) | None | ↑ genotoxicity in vapers |
| Franco et al. (2016) | Cross-sectional | Italy, n=42 | E-cig – Genotoxicity (oral mucosa) | Age, sex | No difference vs non-users |
| Guo et al. (2021) | Cross-sectional | US, n=65 | E-cig – AP sites (oral mucosa) | None | ↓ genotoxicity in vapers |
| Herzog et al. (2024) | Cross-sectional | EU, n=233 | E-cig – Epigenetics (oral mucosa) | None | Similar methylation to smokers |
| Podguski et al. (2022) | Cross-sectional | US, n=12 | E-cig (EVALI) – Genotoxicity & oxidative stress (urine) | None | ↑ genotoxicity, no change in oxidative stress |
| Pop et al. (2021) | Cross-sectional | Romania, n=43 | E-cig – Genotoxicity (oral mucosa) | None | ↑ genotoxicity |
| Sakamaki-Ching et al. (2020) | Cross-sectional | US, n=37 | E-cig – Genotoxicity & oxidative stress (urine) | None | ↑ genotoxicity & oxidative stress |
| Schwarzmeier et al. (2021) | Cross-sectional | Brazil, n=47 | E-cig – Genotoxicity (oral mucosa) | None | ↑ genotoxicity |
| Silveira et al. (2022) | Cohort | US, PATH study, n>10,000 | E-cig – Oral lesions | Demographics, health behaviours | No association |
| Tadin et al. (2024) | Cross-sectional | Croatia, n=120 | E-cig & HTP – Genotoxicity (oral mucosa) | None | ↑ genotoxicity in both groups |
| Tommasi et al. (2023) | Cross-sectional | US, n=48 | E-cig – Genotoxicity (oral mucosa) | Level of use | ↑ in heavy users only |
| Tommasi et al. (2019) | Cross-sectional | US, n=69 | E-cig – Gene expression (oral mucosa) | None | ↓ NOTCH1; altered pathways |
| Emma et al. (2023) | In vitro | Lung fibroblasts, ALI | E-cig – Genotoxicity, mutagenicity | NA | No effect |
| Espinoza-Derout et al. (2019) | In vivo | ApoE-/- mice | E-cig ± nicotine – Liver DNA damage, oxidative stress | NA | ↑ damage with nicotine only |
| Canistro et al. (2017) | In vivo | Rats | E-cig – Genotoxicity, mutagenicity, oxidative stress | NA | ↑ DNA damage, + Ames test |
| Ganapathy et al. (2017) | In vitro | Oral/lung epithelial cells | E-cig – Genotoxicity & oxidative stress | NA | ↑ genotoxicity (nicotine-independent) |
| Hung et al. (2020) | In vitro + in silico | TK6 cells | 150 flavours – Genotoxicity, mutagenicity | NA | 25 in vitro + 46 in silico hits |
| Huynh et al. (2020) | In vivo | NSG mice | E-cig – Lung tumorigenesis | NA | ↑ metastasis, ↓ apoptosis |
| Khalil et al. (2021) | In vitro | A549 cells, ALI | E-cig – Genotoxicity | NA | 3/4 products ↑ DNA damage |
| Lee et al. (2018) | In vivo + in vitro | Mice + human cells | E-cig + NNK – Genotoxicity, tumor transformation | NA | ↑ adducts, ↓ repair, ↑ mutagenesis |
| Lerner et al. (2016) | In vitro | Lung fibroblasts, ALI | E-cig – Genotoxicity | NA | ↑ genotoxicity |
| Ma et al. (2023) | In vivo | Mice | E-cig extract – Genotoxicity | NA | Dose-dependent ↑ 8-oxo-dG |
| Muthumulage et al. (2023) | In vivo | Mice | E-cig ± nicotine – Lung genotoxicity | NA | Nicotine-dependent & independent effects |
| Pearce et al. (2020) | In vitro | Bronchial cells | E-cig – Genotoxicity & oxidative stress | NA | ↑ genotoxicity and ROS |
| Pham et al. (2020) | In vivo | Mice | E-cig – Tumor progression | NA | ↑ growth, metastasis, ↓ apoptosis |
| Platel et al. (2023) | In vivo | Mice | E-cig (18/30W) – Genotoxicity | NA | Only 30W ↑ oxidative DNA damage |
| Rankin et al. (2018) | In vitro | Lung cells | E-cig – Genotoxicity & ROS | NA | No effect |
| Robin et al. (2022) | In vitro | Oral cancer cells | E-cig – Invasion & inflammation | NA | ↑ invasion (flavour-specific) |
| Ruth et al. (2023) | In vitro | A549 cells, ALI | E-cig (0–75W) – Genotoxicity | NA | Only >45W ↑ DNA damage |
| Sun et al. (2021) | In vivo | Mice | E-cig 0–2.4% nicotine – Genotoxicity | NA | ↑ 8-oxo-dG; paradoxical lower damage at 2.4% |
| Sundar et al. (2016) | In vitro | Oral 2D/3D, ALI | E-cig – Genotoxicity & oxidative stress | NA | ↑ DNA strand breaks, ROS |
| Tang et al. (2019) | In vivo | Mice | E-cig – Tumor formation | NA | Lung tumors, bladder hyperplasia |
| Tellez et al. (2023) | In vitro | Bronchial cells | E-cig – Oxidative stress, transformation | NA | ↑ transformation in 1/3 lines |
| Tellez et al. (2021) | In vitro | Oral cells | E-cig (11 e-liquids) – Genotoxicity | NA | 6 products ↑ chromosomal damage |
| Tommasi et al. (2017) | In vitro | Fibroblast cell lines | E-cig – Mutagenicity | NA | No mutagenic effect |
| Yu et al. (2016) | In vitro | Epithelial/cancer cells | E-cig ± nicotine – Genotoxicity | NA | ↑ DNA damage independent of nicotine |
| Zahedi et al. (2018) | In vitro | Lung cancer cells | E-cig – EMT | NA | ↑ motility, EMT markers |
| Zarcone et al. (2023) | In vitro, ALI | Bronchial cells | E-cig vs HTP – Genotoxicity & Oxidative stress | NA | Only HTP ↑ DNA damage |
| HEATED TOBACCO PRODUCTS | | | | | |
| Kawasaki et al. (2021) | Cross-sectional | Japan, n=56 | HTP – Urinary DNA damage markers | Age | ↑ 8-oxo-dG |
| Tadin et al. (2024) | Cross-sectional | Croatia, n=120 | HTP & e-cig – Genotoxicity (oral mucosa) | None | ↑ genotoxicity in both groups |
| Hirata et al. (2022) | In vitro | Lung cancer cells | HTP – EMT, tumorgenesis | NA | ↑ EMT markers and sphere formation |
| Rahman et al. (2022) | In vitro, ALI | Lung mucosal models | HTP – Genotoxicity & Oxidative stress | NA | ↑ 8-oxo-dG, γH2AX, ROS |
| Vivarelli et al. (2021) | In vivo | Rats | HTP – Lung Genotoxicity & Oxidative stress | NA | ↑ oxidative stress, DNA damage |
| Zarcone et al. (2023) | In vitro, ALI | Bronchial cells | HTP vs E-cig – Genotoxicity & Oxidative stress | NA | ↑ damage from HTP only |

WHO, World Health Organization; PAHs, polycyclic aromatic hydrocarbons; VOCs, volatile organic compounds; TSNA, tobacco specific nitrosamines; OR, odds ratio; E-cig, e-cigarette smoke; EMT, epithelial-to-mesenchymal transition; AP site, apurinic/apyrimidinic site; ALI, air-liquid interface; PATH study, US Population Assessment of Tobacco and Health study.

### References for cancer tables

1. Bittoni MA, Carbone DP, Harris RE. Vaping, Smoking and Lung Cancer Risk. J Oncol Res Ther. 2024;9(3):10229.
2. Camila B, Carlos C, Maria-Jose P, Sergio R, Alejandra C, Adriana R. Genotoxicity and hypomethylation of LINE-1 induced by electronic cigarettes. Ecotoxicol Environ Saf. 2023;256:114900.
3. Canistro D, Vivarelli F, Cirillo S, Babot Marquillas C, Buschini A, Lazzaretti M, et al. E-cigarettes induce toxicological effects that can raise the cancer risk. Sci Rep. 2017;7(1):2028.
4. Cheng G, Guo J, Carmella SG, Lindgren B, Ikuemonisan J, Niesen B, et al. Increased acrolein-DNA adducts in buccal brushings of e-cigarette users. Carcinogenesis. 2022;43(5):437-44.
5. Emma R, Fuochi V, Distefano A, Partsinevelos K, Rust S, Zadjali F, et al. Cytotoxicity, mutagenicity and genotoxicity of electronic cigarettes emission aerosols compared to cigarette smoke: the REPLICA project. Sci Rep. 2023;13(1):17859.
6. Espinoza-Derout J, Shao XM, Bankole E, Hasan KM, Mtume N, Liu Y, et al. Hepatic DNA Damage Induced by Electronic Cigarette Exposure Is Associated With the Modulation of NAD+/PARP1/SIRT1 Axis. Front Endocrinol (Lausanne). 2019;10:320.
7. Franco T, Trapasso S, Puzzo L, Allegra E. Electronic Cigarette: Role in the Primary Prevention of Oral Cavity Cancer. Clinical medicine insights Ear, nose and throat. 2016;9:7-12.
8. Ganapathy V, Manyanga J, Brame L, McGuire D, Sadhasivam B, Floyd E, et al. Electronic cigarette aerosols suppress cellular antioxidant defenses and induce significant oxidative DNA damage. PLoS One. 2017;12(5):e0177780.
9. Guo J, Ikuemonisan J, Hatsukami DK, Hecht SS. Liquid Chromatography-Nanoelectrospray Ionization-High-Resolution Tandem Mass Spectrometry Analysis of Apurinic/Apyrimidinic Sites in Oral Cell DNA of Cigarette Smokers, e-Cigarette Users, and Nonsmokers. Chem Res Toxicol. 2021;34(12):2540-8.
10. Herzog C, Jones A, Evans I, Raut JR, Zikan M, Cibula D, et al. Cigarette Smoking and E-cigarette Use Induce Shared DNA Methylation Changes Linked to Carcinogenesis. Cancer Res. 2024;84(11):1898-914.
11. Hirata N, Horinouchi T, Kanda Y. Effects of cigarette smoke extract derived from heated tobacco products on the proliferation of lung cancer stem cells. Toxicol Rep. 2022;9:1273-80.
12. Hung PH, Savidge M, De M, Kang JC, Healy SM, Valerio LG, Jr. In vitro and in silico genetic toxicity screening of flavor compounds and other ingredients in tobacco products with emphasis on ENDS. J Appl Toxicol. 2020;40(11):1566-87.
13. Huynh D, Huang J, Le LTT, Liu D, Liu C, Pham K, et al. Electronic cigarettes promotes the lung colonization of human breast cancer in NOD-SCID-Gamma mice. Int J Clin Exp Pathol. 2020;13(8):2075-81.
14. Kawasaki Y, Li YS, Watanabe S, Ootsuyama Y, Kawai K. Urinary biomarkers for secondhand smoke and heated tobacco products exposure. J Clin Biochem Nutr. 2021;69(1):37-43.
15. Khalil C, Chahine JB, Haykal T, Al Hageh C, Rizk S, Khnayzer RS. E-cigarette aerosol induced cytotoxicity, DNA damages and late apoptosis in dynamically exposed A549 cells. Chemosphere. 2021;263:127874.
16. Lee HW, Park SH, Weng MW, Wang HT, Huang WC, Lepor H, et al. E-cigarette smoke damages DNA and reduces repair activity in mouse lung, heart, and bladder as well as in human lung and bladder cells. Proc Natl Acad Sci U S A. 2018;115(7):E1560-E9.
17. Lerner CA, Rutagarama P, Ahmad T, Sundar IK, Elder A, Rahman I. Electronic cigarette aerosols and copper nanoparticles induce mitochondrial stress and promote DNA fragmentation in lung fibroblasts. Biochem Biophys Res Commun. 2016;477(4):620-5.
18. Ma T, Chen H, Liao YP, Li J, Wang X, Li L, et al. Differential Toxicity of Electronic Cigarette Aerosols Generated from Different Generations of Devices In Vitro and In Vivo. Environ Health (Wash). 2023;1(5):315-23.
19. Muthumalage T, Rahman I. Pulmonary immune response regulation, genotoxicity, and metabolic reprogramming by menthol- and tobacco-flavored e-cigarette exposures in mice. Toxicol Sci. 2023;193(2):146-65.
20. Pearce K, Gray N, Gaur P, Jeon J, Suarez A, Shannahan J, et al. Toxicological analysis of aerosols derived from three electronic nicotine delivery systems using normal human bronchial epithelial cells. Toxicol In Vitro. 2020;69:104997.
21. Pham K, Huynh D, Le L, Delitto D, Yang L, Huang J, et al. E-cigarette promotes breast carcinoma progression and lung metastasis: Macrophage-tumor cells crosstalk and the role of CCL5 and VCAM-1. Cancer Lett. 2020;491:132-45.
22. Platel A, Dusautoir R, Kervoaze G, Dourdin G, Gateau E, Talahari S, et al. Comparison of the in vivo genotoxicity of electronic and conventional cigarettes aerosols after subacute, subchronic and chronic exposures. J Hazard Mater. 2022;423(Pt B):127246.
23. Podguski S, Kaur G, Muthumalage T, McGraw MD, Rahman I. Noninvasive systemic biomarkers of e-cigarette or vaping use-associated lung injury: a pilot study. ERJ Open Res. 2022;8(2).
24. Pop AM, Coros R, Stoica AM, Monea M. Early Diagnosis of Oral Mucosal Alterations in Smokers and E-Cigarette Users Based on Micronuclei Count: A Cross-Sectional Study among Dental Students. Int J Environ Res Public Health. 2021;18(24).
25. Rahman M, Irmler M, Introna M, Beckers J, Palmberg L, Johanson G, et al. Insight into the pulmonary molecular toxicity of heated tobacco products using human bronchial and alveolar mucosa models at air-liquid interface. Sci Rep. 2022;12(1):16396.
26. Rankin GD, Wingfors H, Uski O, Hedman L, Ekstrand-Hammarstrom B, Bosson J, et al. The toxic potential of a fourth-generation E-cigarette on human lung cell lines and tissue explants. J Appl Toxicol. 2019;39(8):1143-54.
27. Robin HP, Trudeau CN, Robbins AJ, Chung EJ, Rahman E, Strickland OLG, et al. Inflammation and Invasion in Oral Squamous Cell Carcinoma Cells Exposed to Electronic Cigarette Vapor Extract. Front Oncol. 2022;12:917862.
28. Ruth T, Daniel J, Konig A, Trittler R, Garcia-Kaufer M. Inhalation toxicity of thermal transformation products formed from e-cigarette vehicle liquid using an in vitro lung model exposed at the Air-Liquid Interface. Food Chem Toxicol. 2023;182:114157.
29. Sakamaki-Ching S, Williams M, Hua M, Li J, Bates SM, Robinson AN, et al. Correlation between biomarkers of exposure, effect and potential harm in the urine of electronic cigarette users. BMJ Open Respir Res. 2020;7(1).
30. Schwarzmeier LAT, da Cruz BS, Ferreira CCP, Carvalho B, Alves MGO, Lima Carta CF, et al. E-cig might cause cell damage of oral mucosa. Oral Surg Oral Med Oral Pathol Oral Radiol. 2021;131(4):435-43.
31. Silveira ML, Everard CD, Sharma E, Lauten K, Alexandridis AA, Duffy K, et al. Tobacco Use and Incidence of Adverse Oral Health Outcomes Among US Adults in the Population Assessment of Tobacco and Health Study. JAMA Netw Open. 2022;5(12):e2245909.
32. Sun YW, Chen KM, Atkins H, Aliaga C, Gordon T, Guttenplan JB, et al. Effects of E-Cigarette Aerosols with Varying Levels of Nicotine on Biomarkers of Oxidative Stress and Inflammation in Mice. Chem Res Toxicol. 2021;34(4):1161-8.
33. Sundar IK, Javed F, Romanos GE, Rahman I. E-cigarettes and flavorings induce inflammatory and pro-senescence responses in oral epithelial cells and periodontal fibroblasts. Oncotarget. 2016;7(47):77196-204.
34. Tadin A, Stazic V, Galic N, Zeljezic D. Evaluation of Cytotoxic and Genotoxic Effects in Buccal Mucosal Cells in Non-Smokers and Users of Traditional Combustible Tobacco Products and Non-Combustible Alternatives. J Xenobiot. 2024;14(1):154-65.
35. Tang MS, Lee HW, Weng MW, Wang HT, Hu Y, Chen LC, et al. DNA damage, DNA repair and carcinogenicity: Tobacco smoke versus electronic cigarette aerosol. Mutat Res Rev Mutat Res. 2022;789:108409.
36. Tang MS, Wu XR, Lee HW, Xia Y, Deng FM, Moreira AL, et al. Electronic-cigarette smoke induces lung adenocarcinoma and bladder urothelial hyperplasia in mice. Proc Natl Acad Sci U S A. 2019;116(43):21727-31.
37. Tellez CS, Grimes MJ, Juri DE, Do K, Willink R, Dye WW, et al. Flavored E-cigarette product aerosols induce transformation of human bronchial epithelial cells. Lung Cancer. 2023;179:107180.
38. Tellez CS, Grimes MJ, Juri DE, Do K, Willink R, Dye WW, et al. Flavored E-cigarette product aerosols induce transformation of human bronchial epithelial cells. Lung Cancer. 2023;179:107180.
39. Tellez CS, Juri DE, Phillips LM, Do K, Yingling CM, Thomas CL, et al. Cytotoxicity and Genotoxicity of E-Cigarette Generated Aerosols Containing Diverse Flavoring Products and Nicotine in Oral Epithelial Cell Lines. Toxicol Sci. 2021;179(2):220-8.
40. Tommasi S, Bates SE, Behar RZ, Talbot P, Besaratinia A. Limited mutagenicity of electronic cigarettes in mouse or human cells in vitro. Lung Cancer. 2017;112:41-6.
41. Tommasi S, Blumenfeld H, Besaratinia A. Vaping Dose, Device Type, and E-Liquid Flavor are Determinants of DNA Damage in Electronic Cigarette Users. Nicotine Tob Res. 2023;25(6):1145-54.
42. Tommasi S, Caliri AW, Caceres A, Moreno DE, Li M, Chen Y, et al. Deregulation of Biologically Significant Genes and Associated Molecular Pathways in the Oral Epithelium of Electronic Cigarette Users. Int J Mol Sci. 2019;20(3).
43. Vivarelli F, Canistro D, Cirillo S, Elias RJ, Granata S, Mussoni M, et al. Unburned Tobacco Cigarette Smoke Alters Rat Ultrastructural Lung Airways and DNA. Nicotine Tob Res. 2021;23(12):2127-34.
44. Yu V, Rahimy M, Korrapati A, Xuan Y, Zou AE, Krishnan AR, et al. Electronic cigarettes induce DNA strand breaks and cell death independently of nicotine in cell lines. Oral Oncol. 2016;52:58-65.
45. Zahedi A, Phandthong R, Chaili A, Remark G, Talbot P. Epithelial-to-mesenchymal transition of A549 lung cancer cells exposed to electronic cigarettes. Lung Cancer. 2018;122:224-33.
46. Zarcone G, Lenski M, Martinez T, Talahari S, Simonin O, Garcon G, et al. Impact of Electronic Cigarettes, Heated Tobacco Products and Conventional Cigarettes on the Generation of Oxidative Stress and Genetic and Epigenetic Lesions in Human Bronchial Epithelial BEAS-2B Cells. Toxics. 2023;11(10).

**Supplemental table 5. Summary of literature review: studies investigating E-cigarettes in relation to cardiovascular outcomes.**

| Study | Design | Population/Model | Exposure & Outcome | Adjusted for | Key Findings (OR/HR/PR/β, 95% CI) |
| --- | --- | --- | --- | --- | --- |
| Epidemiological Studies | | | | | |
| Chen et al. 2024 | Systematic review/meta-analysis | 7 cross-sectional studies (US, n=864,888) | Exclusive e-cig use vs never; CVD composite | NA | OR 1.24 (0.93, 1.67) |
| Glantz et al. 2024 | Systematic review/meta-analysis | 11 cross-sectional & 4 longitudinal (US) | E-cig vs never; CVD composite and stroke | Low risk of bias | CVD: OR 1.24 (1.05, 1.46); Stroke: OR 1.32 (0.98, 1.76) |
| Banks et al. 2023 | Umbrella review | Systematic/meta-reviews (2017–2021) | E-cig vs never; clinically manifest CVD | NA | Insufficient data |
| Travis et al. 2022 | Umbrella review | Systematic/meta-reviews (2020–2022) | E-cig vs never; clinically manifest CVD | NA | Insufficient data |
| Glantz et al. 2024 | Systematic review/meta-analysis | 10 cross-sectional, 1 longitudinal (multi-country) | E-cig vs never; cardiometabolic outcomes | Low risk of bias | OR 1.25 (1.18, 1.33) |
| Lee et al. 2024 | Systematic review/meta-analysis | 4 cross-sectional (US, n=769) | E-cig vs never; flow-mediated dilation (FMD) | Adjusted (not specified) | MD –1.47 (–3.96, 1.02) |
| Experimental Human Studies | | | | | |
| Siddiqui et al. 2023 | Systematic review/meta-analysis | 27 RCTs (n=863, Europe & US) | Acute e-cig exposure; HR, BP, MAP, PWV, CD40L, P-selectin, FMD | NA | HR: WMD 1.22 bpm; SBP: 0.51 mmHg; DBP: 0.62 mmHg; FMD: –1.71 (–2.97, 0.46) |
| Travis et al. 2022 | Umbrella review | Various human and animal studies | Acute/chronic e-cig; HR, BP, endothelial function, oxidative stress | NA | Acute effects likely; chronic effects unclear |
| Banks et al. 2023 | Umbrella review | Various reviews | E-cig; subclinical atherosclerosis, BP, HR | NA | Lack of evidence |
| Animal and In Vitro Studies | | | | | |
| Travis et al. 2022 | Umbrella review | In vitro (n=3), mice (n=2) | E-cig; oxidative stress, endothelial dysfunction, vascular inflammation | NA | ↑ ROS, ↓ antioxidants, endothelial changes, ↑ plaque, angiogenesis |
| Kennedy et al. 2019 | Systematic review | 8 in vitro human cell studies | E-cig 4–72h; oxidative stress, endothelial function, platelet function | NA | ↑ Oxidative stress, ↓ endothelial function, ↑ platelet activation |
| Kennedy et al. 2019 | Systematic review | 5 in vivo animal studies (mice) | E-cig vs air/saline; cardiac, vascular, platelet effects | NA | Cardiac & vascular dysfunction, ↑ platelet aggregation and coagulation changes |

NA, not available; OR, Odd Ratio; MD, mean difference; WMD, weighted mean difference; SMD, standard mean difference; bpm, beat per minute; mmHg, millimetre of mercury; CHD, coronary heart disease; HF, heart failure; MI, myocardial infarction; MS, metabolic syndrome; WC, waist circumference; FMD, flow-mediated dilation; HR, heart rate; BP, blood pressure; SBP, systolic BP; DBP, diastolic BP; MAP: mean arterial pressure; PWV, pulse wave velocity; C-IMT, carotid intima media thickness; CAC, coronary artery calcification; ROS, reactive oxygen species.

### References for cardiovascular disease tables

1. Banks E, Yazidjoglou A, Brown S, Nguyen M, Martin M, Beckwith K, et al. Electronic cigarettes and health outcomes: umbrella and systematic review of the global evidence. The Medical journal of Australia. 2023;218(6):267-75.
2. Chen C, Huo C, Mattey-Mora PP, Bidulescu A, Parker MA. Assessing the association between e-cigarette use and cardiovascular disease: A meta-analysis of exclusive and dual use with combustible cigarettes. Addictive behaviors. 2024;157:108086.
3. Glantz SA, Nguyen N, Oliveira da Silva AL. Population-Based Disease Odds for E-Cigarettes and Dual Use versus Cigarettes. NEJM evidence. 2024;3(3):EVIDoa2300229.
4. Kennedy CD, van Schalkwyk MCI, McKee M, Pisinger C. The cardiovascular effects of electronic cigarettes: A systematic review of experimental studies. Preventive medicine. 2019;127:105770.
5. Lee J, Yao Z, Boakye E, Blaha MJ. The impact of chronic electronic cigarette use on endothelial dysfunction measured by flow-mediated vasodilation: A systematic review and meta-analysis. Tobacco Induced Diseases. 2024;22.
6. Siddiqi TJ, Rashid AM, Siddiqi AK, Anwer A, Usman MS, Sakhi H, et al. Association of Electronic Cigarette Exposure on Cardiovascular Health: A Systematic Review and Meta-Analysis. Current Problems in Cardiology. 2023;48(9).
7. Travis N, Knoll M, Cadham CJ, Cook S, Warner KE, Fleischer NL, et al. Health Effects of Electronic Cigarettes: An Umbrella Review and Methodological Considerations. International Journal of Environmental Research and Public Health. 2022;19(15).

**Supplemental Table 6. Summary of the literature review: Studies investigating the new nicotine products in relation to diabetes, prediabetes, glucose tolerance, insulin resistance and the metabolic syndrome.**

| Study | Design | Population/Model | Exposure & Outcome | Adjusted for | Key Findings (OR/PR/β, 95% CI) |
| --- | --- | --- | --- | --- | --- |
| E-CIGARETTES | | | | | |
| Cai & Bidulescu, 2023 | Cross-sectional (NHANES) | US adults, n=5,101 | E-cig use (current vs. never) & prediabetes, diabetes, HOMA-IR | Demographics, BMI, tobacco, PA, etc. | Current use: Prediabetes 1.14 (0.68-1.92); Diabetes 0.73 (0.30-1.93), HOMA-IR 1.33 (0.77-2.30). |
| Jeong & Kim, 2024 | Cross-sectional (KNHANES) | Korea, n=460,603 | Current vs. non e-cig use and non-smoking & self-reported diabetes | Demographics, BMI, PA | OR: 1.15 (1.01–1.31); Dual: 1.39 (1.22–1.58) |
| Zhang et al., 2022 | Cross-sectional (BRSS) | US adults, n=600,046 | Current vs. never e-cig use (current and former smokers excluded) & prediabetes | Demographics, tobacco, health status | Current: 1.54 (1.17–2.04); Former: 1.13 (1.00-1.29); Dual: 1.14 (0.97-1.34) |
| Cai & Bidulescu, 2023 | Cross-sectional (NHANES) | US, n=5,121 | Current e-cig use vs. never use & metabolic syndrome | Demographics, tobacco use | PR: 1.30 (1.13–1.50) |
| Kim et al., 2020 | Cross-sectional (KNHANES) | Korea, n=14,738 | Current e-cig use vs. never use & prediabetes/diabetes, metabolic syndrome | Demographics, tobacco, alcohol | Prediabetes or diabetes: Current user : 1.05 (0.78-1.40).  METS: 1.27 (0.96–1.69) |
| Orimoloye et al., 2019 | Cross-sectional (NHANES) | US, n=3,415 | Current e-cig use vs. non-use and non-smoking & HOMA-IR, OGTT glucose | Demographics, BMI, alcohol | HOMA-IR: β=0.20 (–0.09–0.49); Glucose: β=–0.05 |
| Wang et al., 2021 | Cross-sectional | US, n=24 | E-cig use & plasma metabolites | NA | Metabolic changes in TCA cycle |
| Chen et al., 2021 | Animal (Balb/c mice) | Overfed & normal mice, Australia | 6-week e-cig vapour; glucose tolerance, PPARγ | NA | No effect on glucose tolerance or insulin sensitivity |
| Lan et al., 2020 | Animal (ApoE KO mice) | Male mice, China | 18-week e-cig (±nicotine) vs cigarettes vs air | NA | Nicotine e-cigs impaired insulin sensitivity, ↑ lipids |
| Lechausseur et al., 2021 | Animal (C57bl/6N mice) | Mice, Canada | Acute & 9-week e-cig (no nicotine) | NA | No effect on glucose/insulin levels |
| Orimoloye et al., 2019 | Animal (C57Bl6/J mice) | Mice, US | 12-week e-cig (±nicotine) | NA | No effect on glucose, insulin, HOMA-IR |
| Ren et al., 2022 | Animal (C57BL/6J mice) | Mice, China | Acute e-cig exposure (0–8h) | NA | ↑ cholesterol, oxidative stress, apoptosis |
| WHITE SNUS | | | | | |
| Liu et al., 2003 | Animal (Zucker rats) | Japan | Oral nicotine & glucose metabolism | NA | ↓ Plasma glucose after insulin load |
| HEATED TOBACCO PRODUCTS | | | | | |
| Hu et al., 2022^1^ | Cross-sectional | Japan, n=40,291 | HTP & prediabetes, diabetes | Demographics, diet, lifestyle | Exclusive: Prediabetes 1.36 (1.25- 1.47), Diabetes 1.68 (1.45- 1.94); Dual: Prediabetes: 1.26 (1.13–1.39). Diabetes 1.93 (1.63–2.29) |

Metabolic syndrome: abdominal obesity, elevated triglycerides, elevated fasting glucose, reduced HDL-cholesterol, high blood pressure. BMI, Body mass index; BRSS, Behavioural Risk Factor Surveillance System; COPD, Chronic Obstructive Lung Disease; Epi, Epidemiological; HbA1c, Haemoglobin A1c; HOMA-IR, Homeostatic Model Assessment of Insulin Resistance; KNHANES, Korean National Health and Nutrition Examination Survey; NHANES, National Health and Nutrition Examination Survey; OGTT, Oral Glucose Tolerance Test; OR, Odds Ratio; PA, Physical Activity; PR, Prevalence Ratio; ROS, Reactive Oxygen Species; sTNF-α, Serum Tumour Necrosis Factor-alpha; TCA, Tricarboxylic Acid; TyG index, Triglyceride-Glucose Index.

### References for diabetes tables

1. Cai J, Bidulescu A. The association between e-cigarette use or dual use of e-cigarette and combustible cigarette and prediabetes, diabetes, or insulin resistance: Findings from the National Health and Nutrition Examination Survey (NHANES). Drug Alcohol Depend. 2023;251:110948. a
2. Cai J, Bidulescu A. Associations between e-cigarette use or dual use of e-cigarette and combustible cigarette and metabolic syndrome: results from the National Health and Nutrition Examination Survey (NHANES). Ann Epidemiol. 2023;85:93-9.e2. b
3. Chen H, Li G, Chan YL, Zhang HE, Gorrell MD, Pollock CA, et al. Differential effects of 'vaping' on lipid and glucose profiles and liver metabolic markers in obese versus non-obese mice. Front Physiol. 2021;12:755124.
4. Hu H, Miyamoto T, Okazaki H, Eguchi M, Shirasaka T, Kochi T, et al. Heated tobacco product use and abnormal glucose metabolism: a working population-based study. Acta Diabetol. 2023;60(3):371-8.
5. Jeong W, Kim S. Impact of electronic cigarette use on the increased risk of diabetes: the Korean Community Health Survey. Epidemiol Health. 2024;46:e2024029.
6. Kim T, Choi H, Kang J, Kim J. Association between electronic cigarette use and metabolic syndrome in the Korean general population: a nationwide population-based study. PLoS One. 2020;15(8):e0237983.
7. Lan K, Zhang G, Liu L, Guo Z, Luo X, Guan H, et al. Electronic cigarette exposure on insulin sensitivity of ApoE gene knockout mice. Tob Induc Dis. 2020;18:68.
8. Lechasseur A, Mouchiroud M, Tremblay F, Bouffard G, Milad N, Pineault M, et al. Glycerol contained in vaping liquids affects the liver and aspects of energy homeostasis in a sex-dependent manner. Physiol Rep. 2022;10(2):e15146.
9. Liu RH, Mizuta M, Matsukura S. Long-term oral nicotine administration reduces insulin resistance in obese rats. Eur J Pharmacol. 2003;458(1-2):227-34.
10. Orimoloye OA, Uddin SMI, Chen LC, Osei AD, Mirbolouk M, Malovichko MV, et al. Electronic cigarettes and insulin resistance in animals and humans: results of a controlled animal study and the National Health and Nutrition Examination Survey (NHANES 2013-2016). PLoS One. 2019;14(12):e0226744.
11. Ren X, Lin L, Sun Q, Li T, Sun M, Sun Z, et al. Metabolomics-based safety evaluation of acute exposure to electronic cigarettes in mice. Sci Total Environ. 2022;839:156392.
12. Wang Q, Ji X, Rahman I. Dysregulated metabolites serve as novel biomarkers for metabolic diseases caused by e-cigarette vaping and cigarette smoking. Metabolites. 2021;11(6):345.
13. Zhang Z, Jiao Z, Blaha MJ, Osei A, Sidhaye V, Ramanathan M Jr, et al. The association between e-cigarette use and prediabetes: Results from the Behavioral Risk Factor Surveillance System, 2016–2018. Am J Prev Med. 2022;62(6):872-7.

Supplemental Table 7. Summary of the literature review-Epidemiological studies investigating E-cigarettes and HTPs in relation to pregnancy outcomes.

| Study | Design | Population | Exposure & Outcome | Adjusted for | Key Findings |
| --- | --- | --- | --- | --- | --- |
| Cardenas 2019 | Cohort | 248 pregnant women, US | E-cig use; SGA | Age, race/ethnicity | E-cig only vs. non-users: RR 3.1 (0.8–11.7); biomarker-verified: RR 5.1 (1.1–22.2). |
| Harlow 2021 | Cohort | Women in US/Canada trying to conceive | E-cig use; time to pregnancy | Demographics, smoking, BMI, mental health, lifestyle | Current vs. never-users: FR 0.85 (0.68–1.07); former: FR 0.89 (0.78–1.00); no stronger effect at higher doses. |
| Cooper 2022 | Ecological | 755 US counties | Vaping restrictions; infant mortality | Maternal and policy factors | Vaping ban vs. no ban: +0.39 deaths per 1000 live births. |
| Hajek 2022 | RCT | 1140 pregnant women, UK | E-cig vs. nicotine patch; birth outcomes | None | E-cig vs. patch: LBW RR 0.65 (0.47–0.90); no other significant differences. |
| Ammar 2023 | Cross-sectional | 190,707 postpartum women, US | E-cig use; SGA, LBW, preterm birth | Extensive maternal factors | E-cig only vs. non-users: preterm birth RR 1.29 (1.00–1.65); LBW RR 1.38 (1.09–1.75); SGA RR 1.04 (0.76–1.44). |
| Kim 2020 | Cross-sectional | 55,251 postpartum women, US | E-cig use; SGA, LBW, preterm birth | Race, age, education, income, prenatal care, smoking | E-cig vs. no use: SGA OR 1.76 (1.04–2.96); LBW OR 1.53 (1.06–2.22); preterm birth OR 1.86 (1.11–3.12). |
| Wang 2020 | Cross-sectional | 31,973 postpartum women, US | E-cig use; SGA, preterm birth | Age, education, BMI, prenatal care, smoking/vaping | E-cig vs. no use: SGA OR 2.4 (1.0–5.7); preterm birth OR 1.2 (0.5–2.7) |
| Regan 2021a | Cross-sectional | 79,176 postpartum women, US | E-cig use (daily/less than daily); SGA, LBW, preterm birth | Age, race, education, prenatal care, smoking | Daily vs. no use: preterm birth PR 1.94 (1.28–2.93); LBW PR 2.00 (1.34–3.00); no SGA link (~0.8); less frequent use: LBW PR 1.76 (1.04–2.65). |
| Regan 2021b | Cross-sectional | 16,022 postpartum smokers, US | E-cig vs. cigarette; SGA, LBW, preterm birth | Age, race, prenatal care, obstetric risk | E-cig vs. cigarette: preterm birth PR 0.85 (0.55–1.31); SGA PR 0.56 (0.29–1.08); LBW PR 0.81 (0.54–1.21). |
| Wen 2023 | Cross-sectional | 10,428 adolescent mothers, US | E-cig use; SGA | Age, race, BMI, health history | E-cig vs. no use: SGA OR 1.68 (0.89–3.18); not significant. |
| Nian 2024 | Cross-sectional | 29,505 postpartum smokers, US | E-cig initiators vs. CC smokers; SGA, LBW, preterm birth | Age, race, education, BMI, prenatal care, smoking history, etc. | E-cig vs. CC smokers: preterm birth OR 0.97 (0.59–1.61), LBW OR 0.70 (0.41–1.21), SGA OR 0.82 (0.52–1.29). |
| Lin 2023 | Cohort | 597 pregnant women, US | E-cig use (none, before, during); high-risk birth, foetal death | Age, education, health, lifestyle factors | E-cig before: high-risk birth OR 1.14 (0.54–2.40); during: OR 1.19 (0.38–3.73); no significant associations. |
| Galanti 2023 | Cohort | 410 infertile women, Italy | HTP use; oocyte quality | None | HTP vs. cigarette users: no sig. difference; e-cig users had slightly more germinal vesicles (0.48 vs. 0.33; p=0.04). |
| Zaitsu 2021 | Cross-sectional | Pregnant/postpartum women, Japan | HTP use (ever vs. never); HDP, LBW | Age, smoking, education, comorbidity | Ever vs. never HTP use: HDP OR 2.78 (0.84–9.15); LBW OR 2.08 (0.80–5.40). |
| Incognito 2024 | Cohort | 642 pregnant women, Italy | HTP use vs. non-smokers; neonatal outcomes | None | Descriptive: preterm 17% HTP users vs. 4% non-smokers; no formal stats reported. |

CC, conventional cigarettes; CI, confidence interval; FR, fecundability ratio; HDP, Hypertensive disorders of pregnancy; LBW, low birth weight; PR, prevalence ratio; RR, relative risk; SGA, small-for-gestational age.

Supplemental table 8. Summary of the literature review of experimental studies investigating the new nicotine products in relation to pregnancy outcomes

| Study | Model | Exposure | Main Findings |
| --- | --- | --- | --- |
| Smith 2015 | C57BL/6J mice | E-cig (with/without nicotine) vs. air | E-cig (with nicotine) reduced pup weight; increased anxiety-like behavior and cognitive changes in adulthood. |
| Lauterstein 2016 | C57BL/6 mice | E-cig (with/without nicotine) vs. air | E-cig (without nicotine) altered gene expression in frontal cortex; linked to neurobiological effects. |
| Chen 2018a | BALB/C mice | E-cig (with/without nicotine), tobacco smoke, air | E-cig (without nicotine) led to highest fat mass and upregulation of brain metabolic genes. |
| Nguyen 2018 | BALB/C mice | E-cig (with/without nicotine) vs. air | E-cig exposure impaired memory, reduced anxiety, and altered brain DNA methylation and gene expression. |
| Chen 2018b | BALB/C mice | E-cig (with/without nicotine) vs. air | E-cig exposure altered weight, fat mass, liver weight, lung inflammation markers, and global DNA methylation. |
| Orzabal 2019 | Sprague-Dawley rats | E-cig (with/without nicotine) vs. air | E-cig (with nicotine) reduced fetal growth and impaired uterine and umbilical artery blood flow. |
| Wetendorf 2019 | C57BL/6J mice | E-cig (with nicotine) vs. air | E-cig exposure delayed litter onset, reduced embryo implantation, and reduced adult offspring weight. |
| Al-Sawalha 2020 | Wistar rats | E-cig (with nicotine) vs. air | E-cig offspring had impaired memory and increased oxidative stress marker (SOD) in hippocampus. |
| Sifat 2020 | CD1 mice | E-cig (with nicotine) vs. air | E-cig reduced pup weight, increased brain injury risk, and impaired motor and cognitive functions. |
| Church 2020 | CD1 mice | E-cig (with/without nicotine) vs. air | E-cig exposure altered stress coping, memory performance, and brain cytokines; nicotine group showed immune changes. |
| Chen 2022 | Sprague-Dawley rats | E-cig vapor vs. air | E-cig disrupted brain calcium signaling, reduced microglia, and increased vulnerability to brain injury. |
| Cahill 2022 | BALB/C mice | E-cig (with nicotine) vs. air | E-cig exposure reduced birthweight, impaired lung development and function, and altered lung gene expression. |
| Archie 2023a | CD1 mice | E-cig (with nicotine) vs. air | E-cig reduced offspring weight, impaired blood-brain barrier markers, and worsened motor and cognitive outcomes. |
| Archie 2023b | CD1 mice | E-cig (with/without nicotine) vs. air | E-cig increased ROS, reduced antioxidant markers, and raised inflammatory markers in neurons. |
| AlHarthi 2023 | C57BL/6 mice | E-cig (with/without nicotine) vs. air | Nicotine-exposed offspring had increased early weight, more locomotor activity, and impaired recognition memory. |
| Awada 2024 | C57BL/6 mice | E-cig (with/without nicotine) vs. air | E-cig increased glucose metabolism proteins and neuroinflammation-related gene expression in offspring brains. |
| Yoshida 2022 | CD1 mice | Heat-not-burn vs. conventional tobacco vs. air | Heat-not-burn exposure impaired testis structure and reduced sperm production at 5 weeks, no hormone changes observed. |

ROS, reactive oxygen species

### References for pregnancy tables

1. AlHarthi A, Alasmari F, AlSharari SD, Alrasheed NM, Alshammari MA, Alshammari TK. Investigating Behavioral and Neuronal Changes in Adolescent Mice Following Prenatal Exposure to Electronic Cigarette (E-Cigarette) Vapor Containing Nicotine. *Brain sciences.* 2023;13(10).
2. Al-Sawalha N, Alzoubi K, Khabour O, et al. Effect of electronic cigarette aerosol exposure during gestation and lactation on learning and memory of adult male offspring rats. *Physiology & behavior.* 2020;221:112911.
3. Ammar L, Tindle HA, Miller AM, et al. Electronic cigarette use during pregnancy and the risk of adverse birth outcomes: A cross-sectional surveillance study of the US Pregnancy Risk Assessment Monitoring System (PRAMS) population. PloS one. 2023;18(10):e0287348.
4. Archie SR, Sifat AE, Mara D, et al. Impact of in-utero electronic cigarette exposure on neonatal neuroinflammation, oxidative stress and mitochondrial function. *Frontiers in pharmacology.* 2023;14:1227145.
5. Archie SR, Sifat AE, Zhang Y, et al. Maternal e-cigarette use can disrupt postnatal blood-brain barrier (BBB) integrity and deteriorates motor, learning and memory function: influence of sex and age. *Fluids and barriers of the CNS.* 2023;20(1):17.
6. Awada C, Saporito AF, Zelikoff JT, Klein CB. E-Cigarette Exposure Alters Neuroinflammation Gene and Protein Expression in a Murine Model: Insights from Perinatally Exposed Offspring and Post-Birth Mothers. *Genes.* 2024;15(3).
7. Cahill KM, Johnson TK, Perveen Z, Schexnayder M, Xiao R, Heffernan LM, et al. In utero exposures to mint-flavored JUUL aerosol impair lung development and aggravate house dust mite-induced asthma in adult offspring mice. Toxicology. 2022 Jul;477.
8. Cardenas VM, Cen R, Clemens MM, et al. Use of Electronic Nicotine Delivery Systems (ENDS) by pregnant women I: Risk of small-for-gestational-age birth. *Tobacco induced diseases.* 2019;17:44.
9. Chen H, Li G, Chan YL, et al. Modulation of neural regulators of energy homeostasis, and of inflammation, in the pups of mice exposed to e-cigarettes. *Neuroscience letters.* 2018;684:61-66.
10. Chen Y, Liu Q, Li W, Deng X, Yang B, Huang X. Association of prenatal and childhood environment smoking exposure with puberty timing: a systematic review and meta-analysis. *Environ Health Prev Med.* 2018;23(1):33.
11. Chen Z, Chen WQ, Li Y, Moos M, Xiao DL, Wang C. Single-nucleus chromatin accessibility and RNA sequencing reveal impaired brain development in prenatally e-cigarette exposed neonatal rats. *iScience.* 2022;25(8):25.
12. Church JS, Chace-Donahue F, Blum JL, Ratner JR, Zelikoff JT, Schwartzer JJ. Neuroinflammatory and Behavioral Outcomes Measured in Adult Offspring of Mice Exposed Prenatally to E-Cigarette Aerosols. *Environmental health perspectives.* 2020;128(4):47006.
13. Cooper M, Pesko MF. The effect of E-cigarette indoor vaping restrictions on infant mortality. *South Econ J.* 2022:44.
14. Galanti F, Licata E, Paciotti G, et al. Impact of different typologies of smoking on ovarian reserve and oocyte quality in women performing ICSI cycles: an observational prospective study. Eur Rev Med Pharmacol Sci. 2023;27(11):5190-5199.
15. Hajek P, Przulj D, Pesola F, et al. Electronic cigarettes versus nicotine patches for smoking cessation in pregnancy: a randomized controlled trial. Nature medicine. 2022;28(5):958-964.
16. Harlow AF, Hatch EE, Wesselink AK, Rothman KJ, Wise LA. Electronic Cigarettes and Fecundability: Results From a Prospective Preconception Cohort Study. American journal of epidemiology. 2021;190(3):353-361.
17. Incognito GG, Grassi L, Palumbo M. Use of cigarettes and heated tobacco products during pregnancy and maternal-fetal outcomes: a retrospective, monocentric study. Archives of gynecology and obstetrics. 2024;309(5):1981-1989.
18. Kim S, Oancea SC. Electronic cigarettes may not be a "safer alternative" of conventional cigarettes during pregnancy: evidence from the nationally representative PRAMS data. BMC pregnancy and childbirth. 2020;20(1):557.
19. Lauterstein DE, Tijerina PB, Corbett K, et al. E-cigarette aerosols induce neurobiological and neurobehavioral alterations in mice exposed during early life. *Environmental and Molecular Mutagenesis.* 2016;57:S81.
20. Lin SY, Wang L, Zhou W, Kitsantas P, Wen X, Xue H. E-cigarette use during pregnancy and its association with adverse birth outcomes in the US. Preventive medicine. 2023;166:107375.
21. Nguyen T, Li GE, Chen H, Cranfield CG, McGrath KC, Gorrie CA. Maternal E-Cigarette Exposure Results in Cognitive and Epigenetic Alterations in Offspring in a Mouse Model. *Chemical research in toxicology.* 2018;31(7):601-611.
22. Nian H, Odland R, Mindlin S, et al. Demographic characteristics, perinatal smoking patterns, and risk for neonatal health complications among pregnant smokers in the United States who begin using electronic cigarettes during pregnancy: A descriptive study using population-based surveillance. Nicotine & tobacco research : official journal of the Society for Research on Nicotine and Tobacco. 2024;26(11):1455-1462.
23. Orzabal MR, Lunde-Young ER, Ramirez JI, et al. Chronic exposure to e-cig aerosols during early development causes vascular dysfunction and offspring growth deficits. *Translational research : the journal of laboratory and clinical medicine.* 2019;207:70-82
24. Regan AK, Bombard JM, O'Hegarty MM, Smith RA, Tong VT. Adverse Birth Outcomes Associated With Prepregnancy and Prenatal Electronic Cigarette Use. Obstetrics and gynecology. 2021;138(1):85-94. A
25. Regan AK, Pereira G. Patterns of combustible and electronic cigarette use during pregnancy and associated pregnancy outcomes. Scientific reports. 2021;11(1):13508. B
26. Sifat AE, Nozohouri S, Villalba H, et al. Prenatal electronic cigarette exposure decreases brain glucose utilization and worsens outcome in offspring hypoxic-ischemic brain injury. *Journal of neurochemistry.* 2020;153(1):63-79.
27. Smith D, Aherrera A, Lopez A, et al. Adult Behavior in Male Mice Exposed to E-Cigarette Nicotine Vapors during Late Prenatal and Early Postnatal Life. *PloS one.* 2015;10(9):e0137953.
28. Wang X, Lee NL, Burstyn I. Smoking and use of electronic cigarettes (vaping) in relation to preterm birth and small-for-gestational-age in a 2016 U.S. national sample. Preventive medicine. 2020;134:106041.
29. Wen X, Liu L, Moe AA, et al. Use of E-Cigarettes and Cigarettes During Late Pregnancy Among Adolescents. JAMA network open. 2023;6(12):e2347407.
30. Wetendorf M, Randall LT, Lemma MT, et al. E-Cigarette Exposure Delays Implantation and Causes Reduced Weight Gain in Female Offspring Exposed In Utero. *Journal of the Endocrine Society.* 2019;3(10):1907-1916.
31. Yoshida S, Ichinose T, Shibamoto T. Effects of Fetal Exposure to Heat-Not-Burn Tobacco on Testicular Function in Male Offspring. *Biological & pharmaceutical bulletin.* 2020;43(11):1687-1692.
32. Zaitsu M, Hosokawa Y, Okawa S, Hori A, Kobashi G, Tabuchi T. Heated tobacco product use and hypertensive disorders of pregnancy and low birth weight: analysis of a cross-sectional, web-based survey in Japan. BMJ open. 2021;11(9):e052976.
